# Hsa_Circ_0001860 Promotes Smad7 to Enhance MPA Resistance in Endometrial Cancer *via* miR-520h

**DOI:** 10.3389/fcell.2021.738189

**Published:** 2021-11-29

**Authors:** Shuang Yuan, Panchan Zheng, Xiao Sun, Judan Zeng, Wenjiao Cao, Wuyuan Gao, Yudong Wang, Lihua Wang

**Affiliations:** ^1^ Department of Gynecologic Oncology, The International Peace Maternity and Child Health Hospital, School of Medicine, Shanghai Jiao Tong University, Shanghai, China; ^2^ Shanghai Municipal Key Clinical Specialty, Shanghai, China; ^3^ Shanghai Key Laboratory of Embryo Original Disease, Shanghai Jiao Tong University, Shanghai, China

**Keywords:** circular RNA, MPA resistance, endometrial cancer, SMAD7, hsa_circ_0001860, miR-520h

## Abstract

**Background:** Medroxyprogesterone acetate (MPA) is one of the most commonly prescribed progestin for the treatment of endometrial cancer (EC). Despite initial benefits, many patients ultimately develop progesterone resistance. Circular RNA (circRNA) is a kind of noncoding RNA, contributing greatly to the development of human tumor. However, the role of circular RNA in MPA resistance is unknown.

**Methods:** We explored the expression profile of circRNAs in Ishikawa cells treated with (ISK/MPA) or without MPA (ISK) by RNA sequencing, and identified a key circRNA, hsa_circ_0001860. Quantitative reverse transcription polymerase chain reaction (qRT-PCR) was used to verify its expression in MPA-resistant cell lines and tissues. CCK8, Transwell, and flow cytometry were used to evaluate the functional roles of hsa_circ_0001860 in MPA resistance. The interaction between hsa_circ_0001860 and miR-520 h was confirmed by bioinformatics analysis, luciferase reporter assay, and RNA pull-down assay.

**Results:** The expression of hsa_circ_0001860 was significantly downregulated in MPA-resistant cell lines and tissues, and negatively correlated with lymph node metastasis and histological grade of EC. Functional analysis showed that hsa_circ_0001860 knockdown by short hairpin RNA (shRNA) promoted the proliferation, inhibited the apoptosis of Ishikawa cells, and promoted the migration and invasion of Ishikawa cells treated with MPA. Mechanistically, hsa_circ_0001860 promoted Smad7 expression by sponging miR-520 h.

**Conclusion:** Hsa_circ_0001860 plays an important role in the development of MPA resistance in EC through miR-520h/Smad7 axis, and it could be targeted to reverse the MPA resistance in endometrial cancer.

## Introduction

Endometrial cancer is one of the most common gynecological tumors in the United States (Siegel et al., 2020). In 2018, endometrial cancer affected 382,069 women worldwide and resulted in 89,929 deaths, and the incidence and mortality have been rapidly increasing in recent years (Chen et al., 2016b; Bray et al., 2018). More than 90% of endometrial cancers occurs in women over 45 years old, and about 6.4% of them are younger than 45 years old (2021) (Cancer of the endometrium-cancer stat facts, 2021). In order to preserve the fertility of young patients, progesterone such as medroxyprogesterone acetate (MPA) and megestrol acetate (MA) is regarded as the first-line drug for conservative treatment (Rodolakis et al., 2015; La Russa et al., 2018). The expression of progesterone receptor (PR), especially progesterone receptor B (PRB), seems to be necessary for progesterone reaction (Dai et al., 2002). In addition, patients in advanced stages who cannot tolerate surgery also receive conservative treatment. Although 70% of the patients respond to MPA initially, 30–40% of them would recur, and 63% of the patients do not respond when they receive MPA treatment again (Ushijima et al., 2007; Chen M. et al., 2016). Using a constructed stable MPA-resistant Ishikawa cell, it has been shown that SIRT1/FoxO1/SREBP-1, as a pathway targeting PR, is involved in the development of progesterone resistance in endometrial cancer cells (Wang et al., 2018), but the molecular mechanism still remains unclear. Therefore, it is of great significance to elucidate the mechanism and therapeutic target of MPA resistance in EC for individualized treatment of patients.

With the rapid development of RNA sequencing technology, a large number of previously known as “junk molecules” of non-coding RNA have been found to play important roles in human diseases, such as long non-coding RNA (lncRNA) and circRNA (Qu et al., 2015). Characterized by covalent closed loop structure, circRNA has neither 5′ end cap nor 3′ end poly (A) tail. Therefore, it is difficult to be degraded by RNase R and relatively stable (Chen et al., 2015; Chen and Yang, 2015). In addition, circRNA also has the characteristics of cell type and tissue specificity, spatio-temporal specificity, and evolutionary conservatism (Shang et al., 2019). Therefore, these characteristics make it a potential and valuable biomarker for the prognosis and diagnosis in various carcinomas, such as hepatocellular carcinoma (Gong et al., 2018), oral squamous cell carcinoma, and gastric carcinoma (Sun et al., 2018; Zhao et al., 2018).

In recent years, more and more studies have shown that circRNA plays important roles in the development of breast cancer and gynecological cancer such as cervical cancer, ovarian cancer, and endometrial cancer (Chen et al., 2018; Sang et al., 2019; Zhao et al., 2019; Ou et al., 2020). Moreover, circRNA regulates biological functions in a variety of ways, such as serving as microRNA sponges, gene transcription regulators, and protein decoys, and directly translating into protein (Han et al., 2018). Among them, miRNA sponge is the most common mechanism. For example, in endometrial cancer, circ_PUM1 can increase Notch3 by sponging miR-13, thus, promoting the development of endometrial cancer (Zong et al., 2020). However, the function of circRNA as miRNA sponge in the resistance of EC to MPA has not been elucidated.

In this study, we validated the differentially expressed circRNA hsa_circ_0001860 in MPA-sensitive ISK and MPA-resistant KLE and ISK^PRB−/−^ cells. Functional test, luciferase reporter assay, and RNA pull-down assay confirmed that hsa_circ_0001860 downregulation enhanced EC resistance to MPA through the miR-520h/Smad7 axis. These findings may provide evidence to regulate MPA resistance of endometrial cancer by targeting the circRNA hsa_circ_0001860 signaling pathway.

## Materials and Methods

### Patients and Samples

Tissue samples and clinical data were collected from 113 endometrial cancer patients who received surgical treatment in the Shanghai International Peace Maternity and Child Health Hospital from December 2013 to December 2019. All patients were diagnosed according to histopathology report from biopsy after surgery, and none of them received chemotherapy or radiotherapy before operation. The tumor stages and histological grades were established in line with the criteria of the Federation International of Gynecology and Obstetrics (FIGO) 2018 staging system. According to PR expression, patients were divided into MPA-sensitive and MPA-resistant groups. All tissue samples were stored at −80°C until use. The study was approved by the medical research ethics committee of the International Peace Maternal and Child Health Hospital, and a written informed consent of all the patients was obtained when collecting specimens.

### Cell Culture

Human EC cell lines including ISK and KLE were obtained from the American Type Culture Collection (ATCC, Manassas, VA, USA). For stable MPA-resistant cell line ISK^PRB−/−^ establishment, lentiviral solution was produced by transfecting pLKD-CMV-EFGP-2A-Puro-U6-PRB shRNA (OBiO Technology, Shanghai, China) in 293 T cells (ATCC, Manassas, VA, USA) by using Opti-MEM (Gibco) and Lipofectamine 2000 (Invitrogen). Infectious viral solution was collected 48 h after transfection. ISK cells were infected at approximately 50% confluence in viral solution supplemented with 5 μg/ml of polybrene (Sigma), followed by selection with puromycin at 1.0 μg/ml (Sigma) for a week. The sequence for PRB shRNA is listed in Additional File 1: Supplementary Table S1. ISK, KLE, and ISK^PRB−/−^ cells were cultured in Dulbecco’s modified Eagle medium (DMEM)/F12 (Gibco) containing 10% fetal bovine serum (Gibco), 100 μg/ml of penicillin, and 100 U/ml of streptomycin (Gibco) at 37°C in a 5% CO_2_ humidified atmosphere. A previous study indicated that there was a positive correlation between the dose-dependent MPA and reducing the growth of parental Ishikawa cells (Zhao et al., 2007). Hence, DMEM/F12 supplemented with 10% fetal bovine serum and MPA (Selleck, USA) were added at 10 μM. The treatment stocks were initially prepared in DMSO (vehicle) with subsequent dilution for experiments of 1:1,000 (for 10 M). The presence of a vehicle at such dilutions has previously been demonstrated to have no effect on cell growth (Zhao et al., 2007).

### RNA Extraction and Quality Control

Three samples from each of the ISK/MPA and ISK cell lines were collected, and tRNA isolation total RNA was isolated by using Trizol reagent (Invitrogen life, USA) following the instructions of the manufacturer. The quantity and quality of the RNA samples were determined using the NanoDrop ND-1000 instrument (Thermo Fisher Scientific, Waltham, MA, USA). Then RNA Integrity and gDNA contamination test were conducted by Denaturing Agarose Gel Electrophoresis. Sequencing library was determined by Agilent 2,100 Bioanalyzer using the Agilent DNA 1000 chip kit (Agilent, part # 5,067–1,504). The isolated RNA was stored at −80°C for further experimental verification.

### Circular RNA RNA-Seq

CircRNA-Seq high-throughput sequencing and subsequent bioinformatics analysis were all performed by Cloud-Seq Biotech (Shanghai, China). The circRNA sequencing library was constructed by the total RNA from each sample. First of all, 5 μg of total RNA was pretreated by CircRNA Enrichment Kit (Cloud-seq Inc., USA). Second, the prepared RNAs were used to construct the RNA libraries. Then libraries were controlled for quality and quantified using the BioAnalyzer 2,100 system. In addition, libraries were denatured as single-stranded DNA molecules, captured on Illumina flow cells, amplified *in situ* as clusters and finally sequenced for 150 cycles on Illumina HiSeq Sequencer according to the instructions.

### Circular RNA RNA-Seq Data Analysis

Paired-end reads were harvested from Illumina HiSeq 4,000 sequencer, and quality controlled by Q30. After 3′ adaptor trimming and low-quality read removing by the Cutadapt software (v1.9.3), the reads were aligned to the reference genome/transcriptome by theSTAR software, and circRNAs were detected and annotated by the DCC softwareThe. CircBase database and circ2Trait disease database were used to annotate the identified circRNA. The junction read counts were normalized, and differentially expressed circRNAs were determined using the edgeR package of the R software. A value of *p* < 0.05 was set as a threshold. GO and pathway enrichment analysis were performed by using the host genes of the differentially expressed circRNA.

### RNA Isolation and Quantitative Real-Time Polymerase Chain Reaction Assays

Total RNA was isolated from ISK, ISK/MPA, KLE, and ISK^PRB−/-^ cells using Trizol reagent (Takara, Dalian, China), and the RNA concentration was determined by NanoDrop ND-2000 (NanoDrop, USA). To quantify the amounts of mRNA and circRNA, 500 ng of RNA was directly reverse transcribed using Prime Script RT Master Mix (Takara, Dalian, China). Reverse transcription of miRNA was performed using a miScript II RT Kit (Qiagen). cDNA was amplified using Hieff® qPCR SYBR Green Master Mix (Yeasen, Shanghai, China). Real-time PCR was conducted with Quant Studio 7 Flex system (Life Technologies, USA) in accordance with the instructions of the manufacturer. Actin was used as the control for the detection of mRNA and circRNA expression levels, while U6 was used as the control for miRNA expression analysis. Cells treated with DMSO were used as reference for relative gene expression analysis. The primer sequences used for qRT-PCR are listed in Additional File 2: Supplementary Table S2. The ΔΔCt method was used for quantification.

### Cell Transfection

The EC cells planted on a six-well plate with 70–80% confluence were transfected using Lipofectamine 2000 (Invitrogen) according to the instructions of the manufacturer. We often used 2–4 μg of plasmid and 100 pmol miRNA mimics and inhibitors to transfect the EC cells planted on a six-well plate. Two shRNA sequences for the hsa_circ_0001860 were used in this study (sh-circ_0001860–2 has the highest inhibition efficiency and sh-circ_0001860 mentioned in the article refers to sh-circ_0001860–2). ShRNA, miR-520 h mimics, or miR-520 inhibitor was designed and synthesized by Gene Pharma (Shanghai, China). The sequences used are listed in Additional File 1: Supplementary Table S1 and Additional File 3: Supplementary Table S3. To overexpress hsa_circ_0001860, the full length of 631 bp of hsa_circ_0001860 cDNA was cloned into vector pEX-3 (pGCMV/MCS/Neo) (Gene Pharma, Shanghai, China).

### *In Silico* Target Prediction and Luciferase Reporter Assay

The potential targets of hsa_circ_0001860 were predicted based on online software including CircBank, CircInteractome, and StarBase (Li et al., 2014; Dudekulay et al., 2016; Liu et al., 2019). Finally, miR-520 h was predicted as a target gene of hsa_circ_0001860. We also calculated the minimum free energy hybridization score for the miR-520 h binding to hsa_circ_0001860 using the RNAhybrid.

For hsa_circ_0001860 and miR-520 h luciferase reporter assay, the hsa_circ_0001860 sequences containing wild-type or mutated miR-520 h binding sites were, respectively, synthesized and inserted into pMIR-REPORT luciferase (OBiO Technology, Shanghai, China). ISK cells were seeded in 24-well plates and co-transfected with miR-520 h mimics (Gene Pharma, Shanghai, China) or NC mimics combined with luciferase reporter using Lipofectamine 2000 (Invitrogen) according to the protocol of the manufacturer. At 48 h after transfection, luciferase reporter assays were conducted using a dual-luciferase reporter assay system (Promega, Madison, WI, USA) according to the instructions of the manufacturer. Relative luciferase activity was normalized to Renilla luciferase activity.

### Biotin-Coupled Probe RNA Pull-Down Assay

The hsa_circ_0001860 probe and Biotin-NC were designed and synthesized by Gene Pharma (Shanghai, China). In brief, 1 × 10^7^ ISK cells were treated with RNA lysis buffer. The probe was incubated with pierce nucleic acid-compatible streptavidin beads for 3 h at 25°C to acquire probe-coated beads. After that, the probe-coated bead mixture was incubated with the cell lysates overnight at 4°C. The RNA complexes binding to the beads were washed with the wash buffer two times and purified by the Trizol Reagent (Takara, Dalian, China). The expression of miR-520 h was detected by qRT-PCR.

### Western Blot Analysis

Treated cells were lysed in RIPA buffer containing protease inhibitor phenylmethanesulfonyl fluoride (Beyotime, Nanjing, China). About 10–20 μg of protein samples was loaded into the 10% sodium dodecyl sulfate-polyacrylamide (SDS-PAGE) gel and subjected to electrophoresis at 120 V, and then transferred to polyvinylidenefluoride (PVDF) membranes (Millipore, Billerica, MA. USA). The membranes were blocked with 5% BSA in TBST buffer and incubated with specific primary antibodies at 4°C overnight. GAPDH was used as a loading control. The primary antibodies and the secondary antibody were diluted with the primary antibody diluent and the secondary antibody diluent, respectively. Detailed information of antibodies and dilution used in this study are provided in Additional File 4: Supplementary Table S4. The next day, membranes were washed for 15 min three times in TBST and incubated with secondary antibodies for 1 h at room temperature. Immunoreactive bands were visualized by an enhanced chemiluminescence (ECL) system and imaged with Amersham Imager 600.

### Cell Proliferation and Cytotoxicity Assay

Transfected ISK, KLE, and ISK^PRB−/−^ cells were cultured in 96-well plates overnight, then the medium was replaced with 100 μl of medium solution containing the MPA (10 μM) or DMSO (control) for 24, 48, 72, and 96 h. The cell proliferation and cytotoxicity were measured using the Cell Counting Kit-8 (CCK8) following the directions of the manufacturer (Yeasen, Shanghai, China). In brief, 10 μl CCK8 solution was added to the 100 μl of medium solution and incubated at 37°C for 1 h. The absorbance values were measured at 450 nm using a SpectraMax 190 microplate reader (Bio-Rad Model 680).

### Migration and Invasion Assays

After transfecting plasmid and miRNA inhibitors, the cells were subsequently starved in culture medium without FBS for 12 h to remove the effect of proliferation. In the Transwell migration and invasion assay, the upper Transwell chambers (8-μm pore) were coated with 50 μl of Matrigel at a dilution of 1:6 (BD Biosciences, San Jose CA, USA). A total of 1 × 10^5^ cells were seeded into the upper chamber of a 24-well chemotaxis chamber with polycarbonate filters (8-μm pore) (Corning Incorporated, Glendale, AZ, USA). DMEM/F12 supplemented with 10% FBS was added to the lower chamber. Then cells planted in the upper chamber were treated with MPA (10 μM) in serum-deprived media for 24 or 48 h. After the treatment, cells on the upper side of the chamber were removed, and cells on the lower side were fixed with 4% paraformaldehyde for 30 min, stained with crystal violet for 30 min, and photographed under a microscope at × 100 magnification. The number of crystal violet-stained cells was counted in five fields from each well at × 200 magnification.

### Apoptosis Assay

Cell apoptosis was detected using an Annexin V-PE/7-AAD Detection Kit (Yeasen, Shanghai, China) according to the instructions of the manufacturer. Briefly, after incubation with MPA (10 μM) or DMSO (control) for 48 h, cells were trypsinized, washed, and resuspended in binding buffer. Next, 5 μl of Annexin V-PE and 10 μl of 7-AAD were added to the cell suspension and incubated in the dark at 4°C for 15 min. A FACScan flow cytometer and FlowJo software (Tree Star Inc., Ashland, OR, USA) were used to analyze the cells.

### Statistical Analysis

All experiments were performed in triplicate. Data were analyzed with the SPSS software (version 19.0) (SPSS, Inc., Chicago, IL, USA) and presented as the mean ± SD. The statistical significance of the results was calculated using the independent-sample *t*-test or one-way ANOVA test. Clinicopathological features were analyzed by an χ2 test. The interaction between variables was analyzed by Pearson correlation analysis. A *p*-value <  0.05 was considered statistically significant.

## Results

### Identification of Dysregulated Circular RNAs in Ishikawa Cells Upon the Medroxyprogesterone Acetate Treatment

The concentration and purity of total RNAs from different samples were determined by NanoDrop ND-1000 (Thermo Fisher Scientific, Waltham, MA, USA). All RNA samples showed an OD A260/280 ratio between 1.8 and 2.1. Principal component analysis (PCA) of these sequenced samples showed that there was a clear separation relationship between the treated cells and control cells (Additional File 5: Supplementary Figure S1). RNA-seq analysis showed that a total of 4,814 circRNAs were detected. Among them, 964 circRNAs were novel according to the published studies. We then compared the expression profiles of circRNAs between MPA-treated ISK cells and control cells. The results showed 87 differentially expressed circRNAs in MPA-treated ISK cells compared with control cells (|fold change| ≥ 2.0, *p* < 0.05), and among them, 46 were upregulated and 41 were downregulated. It is reported that circRNA is divided into exotic circular RNA (ecircRNA), circular intronic RNA (ciRNAs), and exonic-intron circular RNA (EiciRNA) (Qu et al., 2015). In this study, all circRNAs are located in exons. Moreover, we conducted a clustering analysis of 87 differentially expressed circRNAs (Additional File 6: Supplementary Figure S2 and Additional file 7: Supplementary Table S5).

### Gene Ontology and Kyoto Encyclopedoa of Genes and Genomes Pathway Analysis and Validation of the Expression Levels of Circular RNAs

**FIGURE 1:**
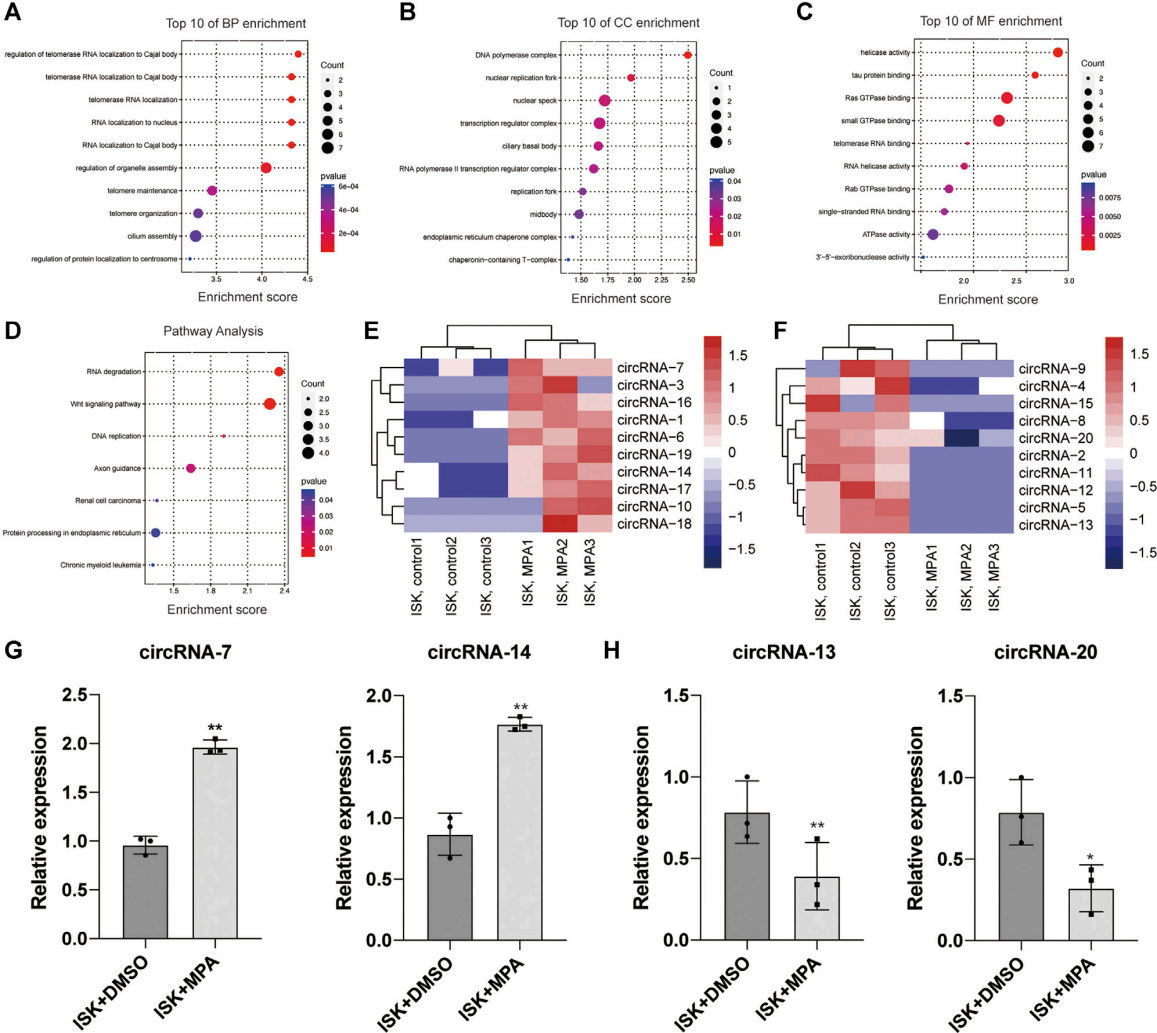
Bioinformatic analysis of circRNA expression pattern in Ishikawa (ISK) cell lines treated with medroxyprogesterone acetate (MPA) compared with untreated. **(E,F)** Heat map evaluation of the circular RNA (circRNA) expression patterns among cells treated with MPA and untreated cells. Each column represents the expression profile of a cell line sample, and each row corresponds to a circRNA. “Red” indicates higher expression level, and “blue” indicates lower expression level. **(A–D)** Biological process (BP), cellular component (CC), and molecular function (MF) terms for the parental genes of dysregulated circRNAs. **(F)** Kyoto Encyclopedia of Genes and Genomes (KEGG) pathways for the parental genes of dysregulated circRNAs. **(G,H)** Validation of the differentially expressed circRNAs by qRT-PCR assay. *p < 0.05, **p < 0.01, ***p < 0.001.

Gene Ontology (GO) includes biological process classification (BP), cellular component (CC), and molecular function classification (MF). Some studies have pointed out that circRNAs may affect the expression of its parental genes, so the function of circRNAs may be related to the function of its parental gene (Bolisetty and Graveley, 2013; Boeckel et al., 2015; Goel et al., 2020; Mehta et al., 2020). Here, the function of the parental gene is used as a prediction of the function of circRNAs. GO terms with a *p*-value <0.05 were selected and ranked by enrichment score [−log10 (*p*-value)]. The top 10 GO analyses were identified according to the enriched dysregulated circRNAs. Among the BP terms, telomerase RNA localization and telomere maintenance have been reported to be associated with tumor (Figure 1A). In MF terms, tau protein binding and ATPase activity might play a role in tumors (Figure 1C). The KEGG analysis showed that the dysregulated circRNAs were mainly enriched in RNA degradation, Wnt signaling pathway, DNA replication, renal cell carcinoma, and chronic myeloid leukemia (Figure 1D).

In order to confirm the RNA-seq data, the 20 most obvious differentially expressed circRNAs were selected for further study, including 10 upregulated circRNAs and 10 downregulated circRNAs (Figures 1E,F and Additional File 8: Supplementary Table S6). We validated their expression levels by qRT-PCR (Figures 1G,H and additional file 9: Supplementary Figure S3). The qRT-PCR analysis revealed that the expression of these circRNAs showed either the same upregulation pattern or the same downregulation pattern as the RNA-seq data. Particularly, hsa_circ_0001860 (circRNA-7) and hsa_circ_0001116 (circRNA-14) were upregulated in the ISK cell line treated with progesterone compared with untreated cells, while hsa_circ_0046843 (circRNA-13) and hsa_circ_0000847 (circRNA-20) were downregulated, which is consistent with our RNA-seq results (Figures 1G,H). These findings indicated that the results of qRT-PCR were well consistent with RNA-seq results, suggesting the high reliability of the RNA-seq expression results.

### Hsa_circ_0001860 is Downregulated in Medroxyprogesterone Acetate-Resistant Endometrial Cancer Cells and Tissues and Negatively Correlated With Lymph Node Metastasis and Histological Grade

**FIGURE 2:**
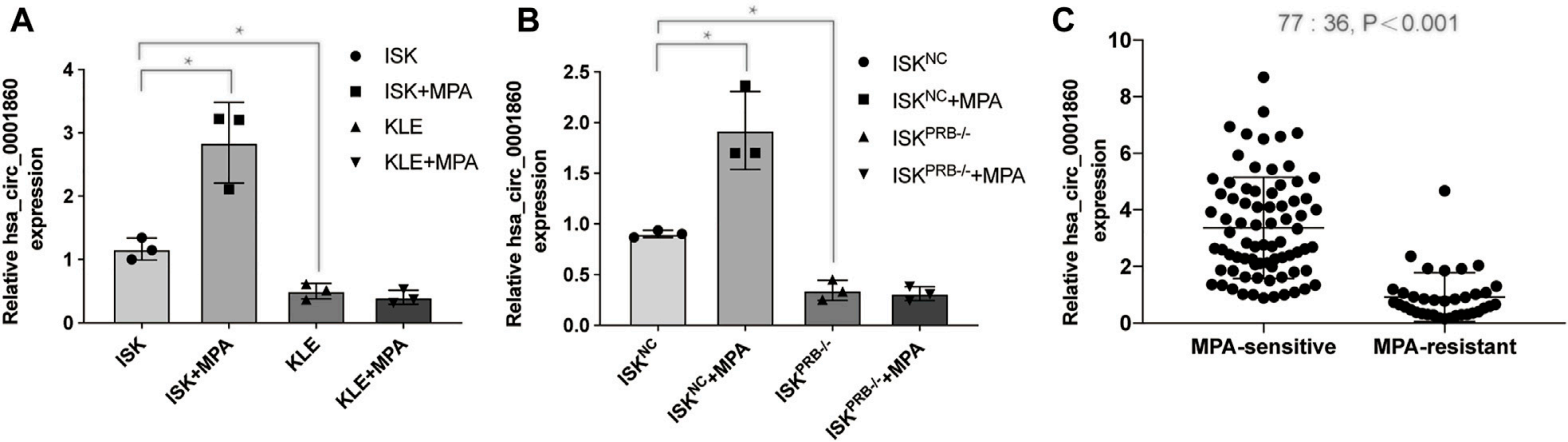
Hsa_circ_0001860 expression is decreased in MPA-resistant EC cells and tissues **(A), (B)** Expression levels of hsa_circ_0001860 in MPA-sensitive EC cell lines (ISK and ISK^NC^) and MPA-resistant EC cell lines (ISK^PRB−/−^ and KLE) treated with dimethyl sulfoxide (DMSO) or MPA. **(C)** qRT-PCR assay showed the expression level of hsa_circ_0001860 in tissue of patients from 36 MPA-resistant EC patients and 77 MPA-sensitive EC patients. *p < 0.05, **p < 0.01, ***p < 0.001.

Among the 20 circRNAs, we chose the most interesting one, hsa_circ_000186, for further study. The circRNA was treated with RNase R, and qPCR analyses were used to compare the gene expression level changes pre- and post-treatment. In contrast to the >10,000-fold change of control GAPDH, hsa_circ_0001860 had less than a twofold change, proving that it is circle RNA (Additional File 10: Supplementary Table S7). In addition, Sanger sequencing was performed to determine the cyclization site of hsa_circ_0001860 (Additional File 11). To further explore the role of hsa_circ_0001860 in progesterone resistance, we used qRT-PCR to verify its expression in MPA-resistant cell lines and tissues. Our data showed that hsa_circ_0001860 was highly expressed in MPA-sensitive EC cell lines (ISK) compared with MPA-resistant EC cell lines (ISK^PRB−/−^ and KLE). Its expression was dramatically increased in ISK by MPA treatment; however, it was not changed by MPA in ISK^PRB−/-^ and KLE (Figures 2A,B). Moreover, the expression of hsa_circ_0001860 was significantly decreased in MPA-resistant EC tissues compared with MPA-sensitive EC tissues (Figure 2C). To explore the correlation between hsa_circ_0001860 expression and clinicopathological parameters, the median hsa_circ_0001860 expression value was used as the cutoff threshold to categorize all patients with EC. The results showed that the level of hsa_circ_0001860 was negatively correlated with stage, histological grade, and lymph node metastasis (Table 1). We also performed multivariate analysis and found that histological grade may affect the expression of hsa_circ_0001860 (Table 2).

**TABLE 1:** Correlation of relative hsa_circ_0001860 expression with the clinicopathological characteristics of 113 patients with endometrial cancer. Note. *p < 0.05, **p < 0.01, ***p < 0.001.

Variables	No. of patients	hsa_circ_0001860 expression		P-value
		Low	High	
Age (years)				
<50	27	11	16	0.2936
≥50	86	45	41	
Stage				
Ⅰ+Ⅱ	92	39	53	0.0014 **
Ⅲ+Ⅳ	21	17	4	
histological grade				
G1+G2	83	33	50	0.0005 ***
G3	30	23	7	
Myometrial invasion				
<1/2	78	36	42	0.28
≥1/2	35	20	15	
Lymph node metastasis				
No	96	42	54	0.0033 **
Yes	17	14	3	

**TABLE 2:** Multiple regression analysis of influencing gene expression. *p < 0.05, **p < 0.01, ***p < 0.001.

Variables	β	SE	95% confidence	t	P-value
Age (years)	0.004	0.018	−0.032 to 0.039	0.216	0.829
Stage	−0.037	0.442	−0.914 to 0.839	0.085	0.933
histological grade	−0.633	0.244	−1.116 to −0.150	2.600	0.011*
Myometrial invasion	−0.055	0.453	−0.953 to 0.843	0.121	0.904
Lymph node metastasis	−1.101	0.861	−2.808 to 0.607	1.278	0.204

### Downregulation of hsa_circ_0001860 Promotes Proliferation and Inhibits Apoptosis of Endometrial Cancer Cells

**FIGURE 3:**
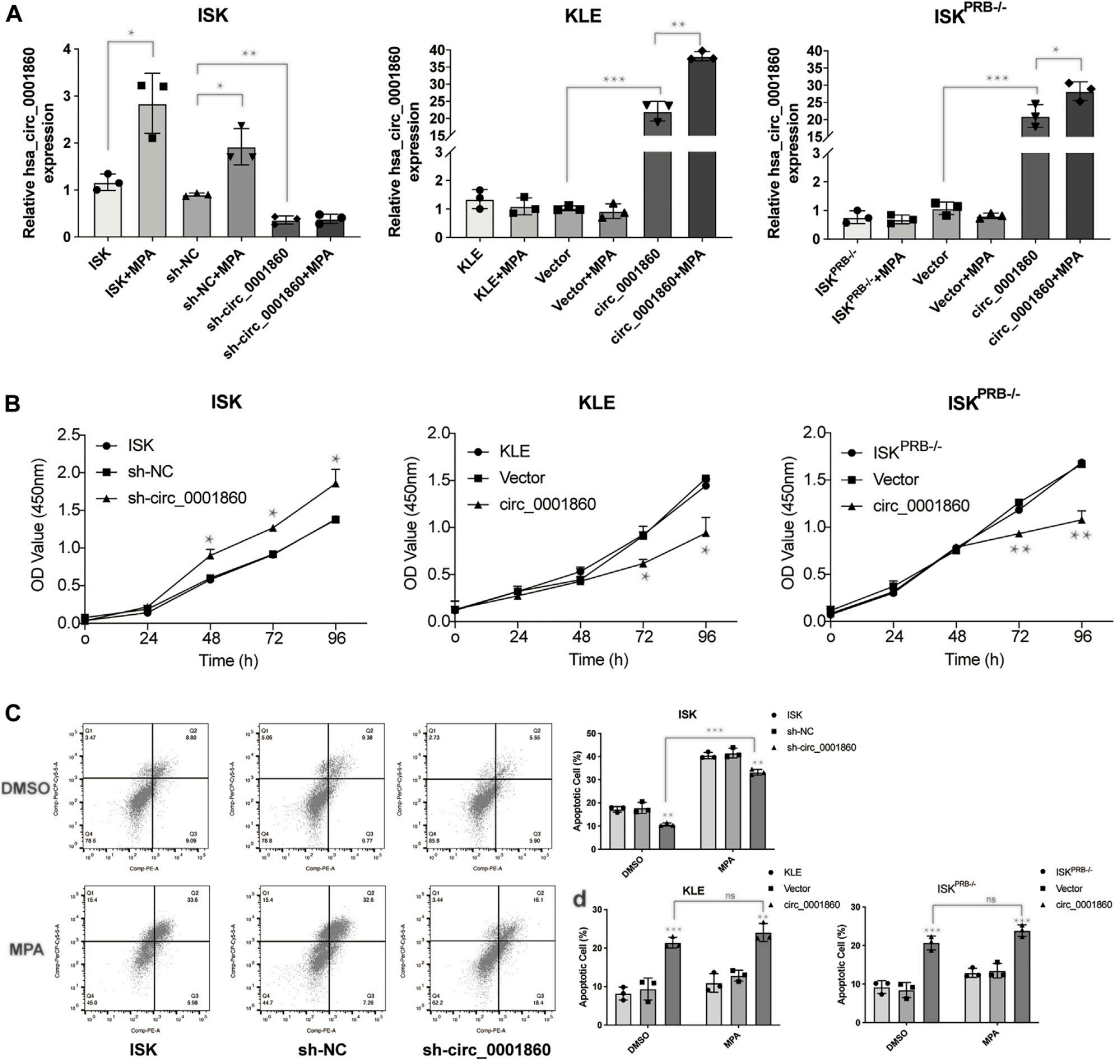
The effects of hsa_circ_0001860 on proliferation and apoptosis of endometrial cancer cells. **(A)** qRT-PCR analysis of hsa_circ_0001860 in ISK, ISK^PRB−/−^, and KLE (blank control), transfected with sh-NC, sh-circ_0001860, vector and circ_0001860 treated with DMSO or MPA. **(B)** Cell counting kit-8 (CCK8) assay was conducted to evaluate cell proliferation. **(C,D)** Cells were treated with MPA (10 μM) or DMSO (control) and subjected to Annexin V-PE/7-AAD staining to detect apoptosis by flow cytometry. *p < 0.05, **p < 0.01, ***p < 0.001.

Given that hsa_circ_0001860 was downregulated in MPA-resistant cell and tissue, we next examined the effect of hsa_circ_0001860 knockdown on EC cell lines, which were transfected with short hairpin RNA (sh-circ_0001860) or the vector control (sh-NC). After transfection, the expression of hsa_circ_0001860 was dramatically decreased in sh-circ_0001860-transfected cells compared with sh-NC-transfected cells, indicating the successful knockdown by hsa-circ_0001860 (Figure 3A). Moreover, the expression of hsa_circ_0001860 was not changed by MPA in sh-circ_0001860-transfected cells, but its expression was significantly increased in ISK^PRB−/−^ and KLE by MPA treatment after the overexpression of hsa_circ_0001860. It was further revealed that the proliferation was increased, and the apoptosis was inhibited when circ_0001860 was knocked down (Figures 3B,C). In line with this, the overexpression of hsa_circ_0001860 in ISK^PRB−/−^ and KLE cells decreased the proliferation and promoted apoptosis (Figures 3B,D). However, little change in MPAsensitivity was observed in these functional assays after downregulating hsa_circ_0001860 in ISK cells or upregulating hsa_circ_0001860 in ISK^PRB−/−^ and KLE cells. ISK cells were still sensitive to MPA treatment, while ISK^PRB−/−^ and KLE cells were resistant to MPA treatment in terms of the proliferation and apoptosis (Figure 3D and Additional File 12: Supplementary Figure S4).

### Downregulation of hsa_circ_0001860 Abolishes Medroxyprogesterone Acetate -Sensitivity in Migration and Invasion of Endometrial Cancer Cells

**FIGURE 4:**
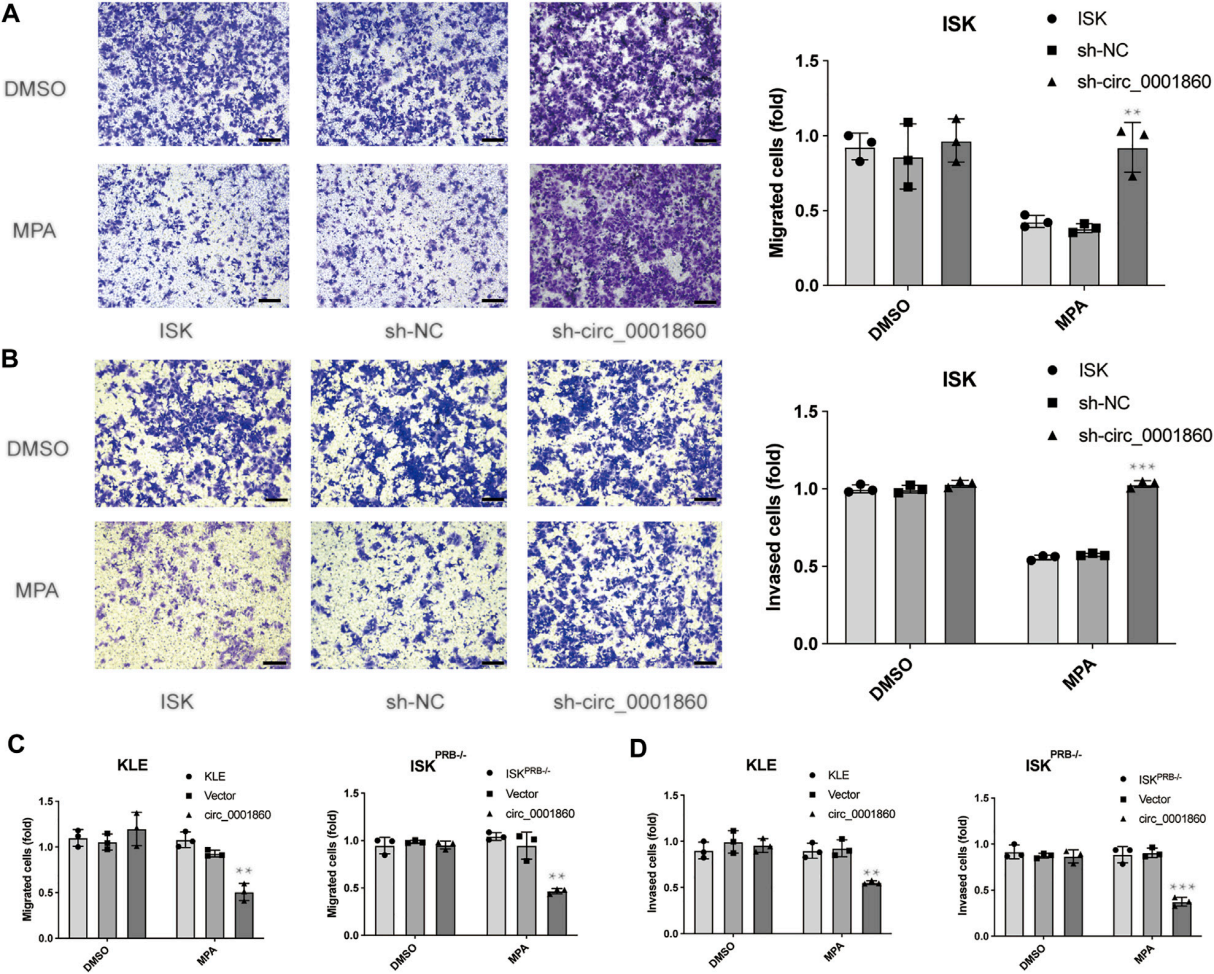
The effect of hsa_circ_0001860 on MPA sensitivity in migration and invasion of endometrial cancer cells. **(A)** Representative pictures and bar graphs showing the effect of hsa_circ_0001860 knockdown on MPA-induced alteration of ISK migration (magnification, × 100; scale bars = 100 μm). **(B)** Representative pictures and bar graphs showing the effect of hsa_circ_0001860 knockdown on MPA-induced alteration of ISK invasion (magnification, × 100; scale bars = 100 μm). **(C, D)** Overexpression of hsa_circ_0001860 in MPA-resistant ISK^PRB−/−^ and KLE restored MPA sensitivity. *p < 0.05, **p < 0.01, ***p < 0.001.

Cell migration and invasion experiments showed that MPA could significantly inhibit the migration and invasion of MPA-sensitive EC cell lines (ISK) compared with DMSO, whereas no effect was observed on MPA-resistant EC cell lines (ISK^PRB−/−^ and KLE). In order to further explore the effect of hsa_circ_0001860 on MPA sensitivity in the migration and invasion of EC cells, we downregulated hsa_circ_0001860 in ISK cells and upregulated hsa_circ_0001860 in ISK^PRB−/−^ and KLE cells. We found that the downregulation of hsa_circ_0001860 abolished MPA-induced reduction of the migration and invasion of ISK cells (Figures 4A,B). On the other hand, overexpression of hsa_circ_0001860 in MPA-resistant ISK^PRB−/−^ and KLE cells inhibited MPA-induced migration and invasion (Figures 4C,D). These results showed that EC cell sensitivity to MPA was mediated by hsa_circ_0001860.

### Hsa_circ_0001860 May Regulate Tumor Progression and Medroxyprogesterone Acetate Sensitivity of Endometrial Cancer Cells *via* Binding to miR-520h

**FIGURE 5:**
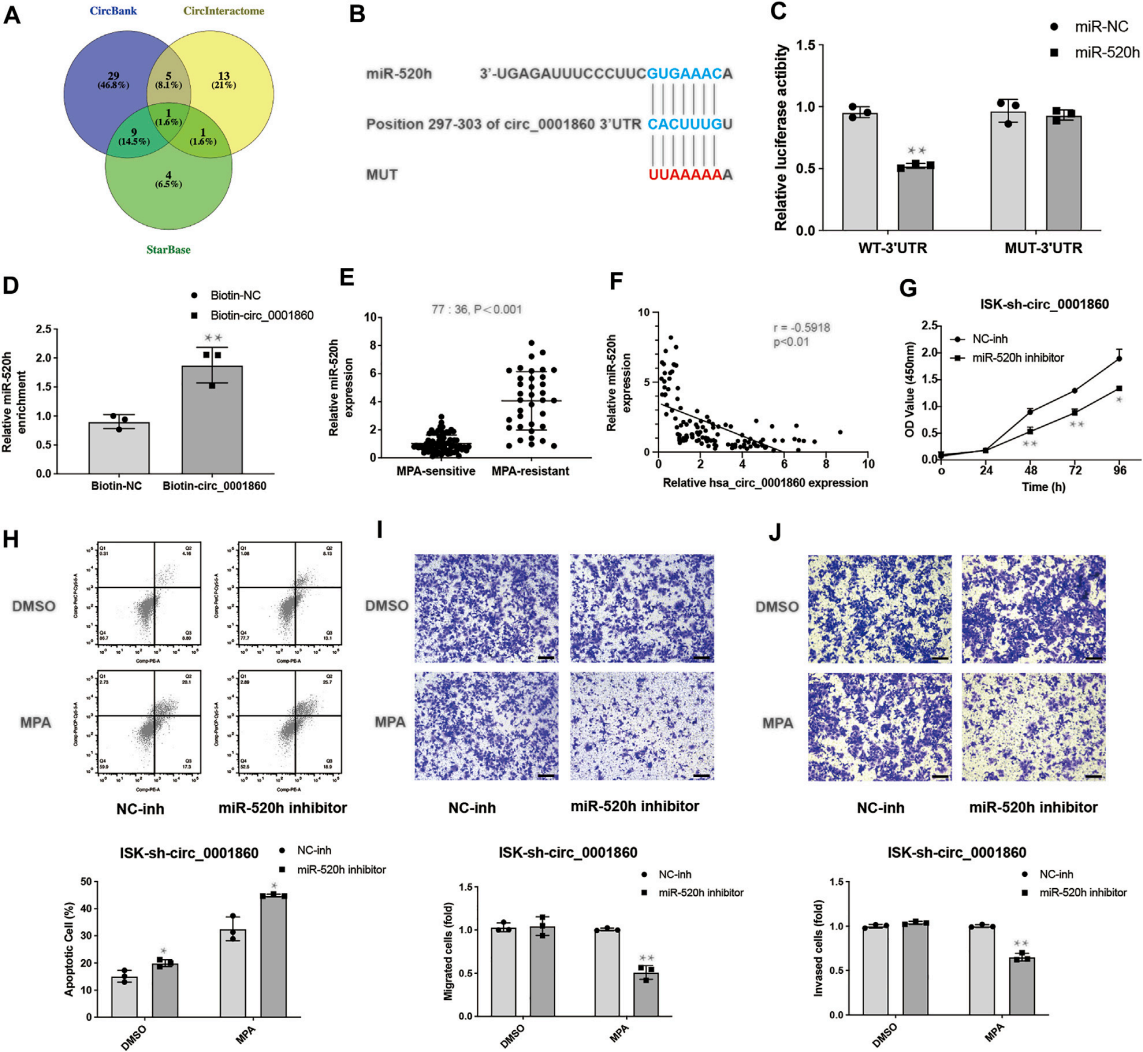
Hsa_circ_0001860 regulates tumor progression and MPA sensitivity of EC cells *via* binding to miR-520 h **(A)** Venn diagram showing genes that are putative hsa_circ_0001860 targets computationally predicted by three algorithms (CircBank, CircInteractome, and StarBase). **(B)** Bioinformatics analysis predicted the presence of a binding site for hsa_circ_0001860 on miR-520 h. **(C)** The relative luciferase activities were analyzed in ISK cells co-transfected with miR-520 h mimics or miR-NC and luciferase reporter WT or MUT. **(D)** The RNA pull-down assay was executed in ISK cells to detect the enrichment of miR-520 h. **(E)** qRT-PCR assay showed the expression level of miR-520 h in tissue of patients from 36 MPA-resistant EC patients and 77 MPA-sensitive EC patients. **(F)** The expression correlation between has_circ_0001860 and miR-520 h. **(G)** Cell proliferation, **(H)** apoptosis, **(I)** migration, and **(J)** invasion of ISK cells after transfection with sh-circ_0001860 combined with miR-520 h inhibitors or NC inhibitor (magnification, × 100; scale bars = 100 μm). *p < 0.05, **p < 0.01, ***p < 0.001.

We next tried to predict the potential targets of hsa_circ_0001860 using the CircBank, CircInteractome, and StarBase (Figure 5A) and identified miR-520 h as a potential target gene of hsa_circ_0001860, which has a binding site for miR-520 h (Figure 5B). We also calculated the minimum free energy hybridization score for the miR-520 h binding to hsa_circ_0001860 using the RNA hybrid (Additional File 13: Supplementary Table S8). Luciferase reporter assay demonstrated that miR-520 h expression significantly reduced the luciferase activity of the reporter in ISK cells co-transfected with WT but not MUT, suggesting that hsa_circ_0001860 may function as a sponge for miR-520 h (Figure 5C). Furthermore, we designed a biotinylated circ_0001860 probe and applied an RNA pull-down assay to confirm the direct interaction between has_circ_0001860 and miR-520 h. The result showed that biotin-labeled circ_0001860 probe captured more miR-520 h compared with the control probe (Figure 5D).

As shown in Figure 5E, miR-520 h was highly expressed in MPA-resistant EC tissues relative to that in MPA-sensitive EC tissues. Besides, the expression of miR-520 h was inversely correlated with has_circ_0001860 level in EC tissues (Figure 5F). Then we investigated the biological functions of miR-520 h by knocking down miR-520 h with miR-520 h inhibitor in ISK cells transfected with sh_circ_0001860. It was found that miR-520 h inhibitor can reverse the effects of sh_circ_0001860 on promoting ISK cell proliferation and inhibiting its apoptosis (Figures 5G,H). Furthermore, knockdown of miR-520 h in sh_circ_0001860-transfected ISK cells rendered them sensitive to MPA as evidenced by the inhibitory effect of MPA on the migration and invasion of miR-520 inhibitor-treated cells (Figures 5I,J).

### Hsa_circ_0001860 Regulates Smad7 Expression and Activates the Smad7/Epithelial-To-Mesenchymal Transition Signaling Pathway

**FIGURE 6:**
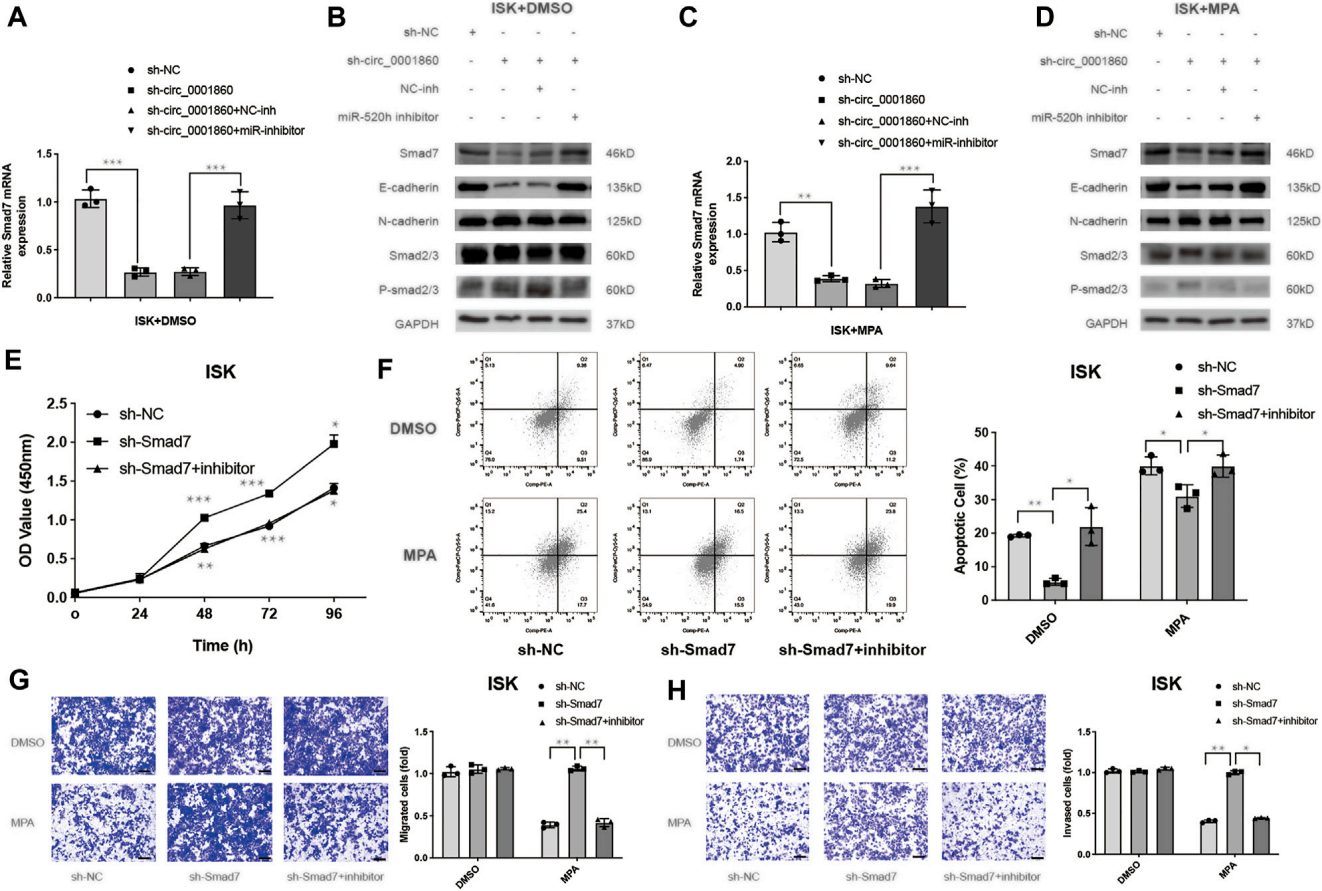
Hsa_circ_0001860 regulates Smad7 expression and activates the Smad7/epithelial-to-mesenchymal transition (EMT) signaling pathway. **(A, C)** The expression levels of Smad7 were analyzed using RT-qPCR. ISK cells were transfected with indicated vectors alone or co-transfected with inhibitors treated with DMSO (control) or MPA (10 μM). **(B, D)** The expression levels of Smad7, Smad7/EMT signaling molecules such as phosphorylated Smad2/3, E-cadherin, and N-cadherin were determined using Western blotting in ISK cells transfected with the indicated vectors alone or co-transfected with inhibitors treated with DMSO (control) or MPA (10 μM). **(E)** Cell proliferation, **(F)** apoptosis, **(G)** migration, and **(H)** invasion of ISK cells after transfection with sh-Smad7 combined with miR-520 h inhibitors or NC inhibitor (magnification, ×100; scale bars = 100 μm). *p < 0.05, **p < 0.01, ***p < 0.001.

It has been reported that miR-520 h enhances EOC cell dissemination and induces EMT *in vivo* by suppressing Smad7 expression (Zhang et al., 2018). We hypothesized that hsa_circ_0001860 could regulate tumorigenesis, migration, and invasion of EC cells mediated by MPA by promoting Smad7 expression *via* acting as a sponge for miR-520 h. We examined the effect of hsa_circ_0001860 on the levels of downstream protein Smad7 of miR-520 h using Western blotting and found that knockdown of hsa_circ_0001860 decreased the levels of Smad7 and influenced EMT signaling pathway-related proteins such as phosphorylated Smad2/3, E-cadherin, and N-cadherin. Meanwhile, concurrent knockdown of miR-520 h and hsa_circ_0001860 reversed hsa_circ_0001860 knockdown-induced decrease in Smad7 expression (Figures 6A,B). Similar results are shown in ISK cells treated with MPA (10 μM) (Figures 6C,D). The densitometric analysis of all Western blots is shown in Additional File 14: Supplementary Figure S5. Moreover, we found that the expression of Smad7 was decreased in MPA-resistant EC tissues compared with MPA-sensitive EC tissues. Besides, the expression of Smad7 was negatively related to miR-520 h level and positively correlated with has_circ_0001860 level in EC tissues (Additional File 15: Supplementary Figure S6).

Previous studies have confirmed that Smad7 rescued the inhibitory effect of MCTP1-AS1 in EC cells (Gao et al., 2021); however, it is unclear whether Smad7 is associated with MPA resistance. We performed experiments and found that downregulation of Smad7 promoted proliferation and inhibits apoptosis (Figures 6E,F). Moreover, Smad7 knockdown abolished MP -sensitivity in migration and invasion of ISK cells (Figures 6G,H). These biological functions also verified that miR-520 h-dependent Smad7 suppression is conserved in the ISK cells (Figures 6E–H). All these results suggest that circRNA hsa_circ_0001860 functions as a ceRNA to regulate Smad7 expression, activate the Smad7/EMT signaling cascade, and promote MPA sensitivity by targeting miR-520 h.

## Discussion

EC is one of the most common gynecologic malignancies. Progestin therapy drugs including MPA and MA are often used to preserve fertility for young patients. However, almost a third of these patients eventually developed MPA resistance (Chen et al., 2016a). There are several mechanisms that underlie the acquired resistance to MPA, such as PR dysregulation, immune system and inflammatory response, and the activation of lipid metabolism (Qiu et al., 2013; Zhao et al., 2013; Li et al., 2019). Recently, some studies show that ncRNAs, such as miRNAs and long ncRNAs (lncRNAs), also play vital roles in MPA resistance. For example, HOTAIR and LSD1 collaboratively repress PRB expression and, thus, reduce progesterone sensitivity in endometrial carcinoma cells (Chi et al., 2019). CHOP and Lnc-CETP-3 might be involved in progesterone-PRB pathway to activate ER stress and provide therapeutic targets for EC patients with negative PRB expression (Cao et al., 2019). However, there is little research focus on the role of circRNA played in MPA resistance of endometrial cancer. Our results identified a novel circRNA hsa_circ_0001860 as being associated with MPA resistance, which could be used to elucidate its underlying regulatory mechanisms in endometrial cancer.

CircRNA has been considered to be essential in the carcinogenesis and tumor progression of EC (Zong et al., 2020). Besides, some studies also suggest that it could be a biomarker candidate for diagnosis and treatment of EC (Xu et al., 2018; Ye et al., 2019). In this study, we demonstrated that circRNA expression is associated with MPA resistance in EC. We identified a novel circRNA hsa_circ_0001860 that was downregulated in tissue samples from MPA-resistant patients and in MPA-resistant cell lines (ISK^PRB−/−^ and KLE). The expression of hsa_circ_0001860 was negatively correlated with histological grade and lymphatic metastasis, suggesting that hsa_circ_0001860 could serve as a diagnostic and therapeutic target for EC. Moreover, downregulation of hsa_circ_0001860 by shRNA accelerated proliferation and decreased apoptosis, and promoted MPA-induced migration and invasion in ISK cells, whereas there was an opposite change in the KLE and ISK^PRB−/−^ cells. Thus, hsa_circ_0001860 may serve as a tumor suppressor and important regulator in MPA-resistant and aggressive EC.

CircRNA can participate in biological functions in a variety of ways, and the most common way is acting as miRNA “sponges” and regulate the expression and activity of the target genes (Han et al., 2018). It is known that miRNAs participate in a majority of biological processes *via* regulating target gene expression (Pu et al., 2019). MiR-520 h has also been studied in various cancers. A recent study shows that miR-520 h promotes the drug resistance of human breast cancer cells through protecting cells from paclitaxel-induced apoptosis by targeting death-associated protein kinase 2 (DAPK2) (Su et al., 2017). In addition, miR-520 h also promotes EOC progression by downregulating Smad7 and activating the TGF-β signaling pathway (Zhang et al., 2018). Smad7 inhibits the TGF-β/Smad signal pathway by preventing the formation of Smad2/4 complex and nuclear translocation after phosphorylation of Smad2 and Smad3, thus, inhibiting EMT (Gonzalez and Medici, 2014). Therefore, we speculate that hsa_circ_0001860 might act as an miRNA sponge for miR-520h, thus, affecting MPA resistance through the EMT signaling pathway.

**FIGURE 7:**
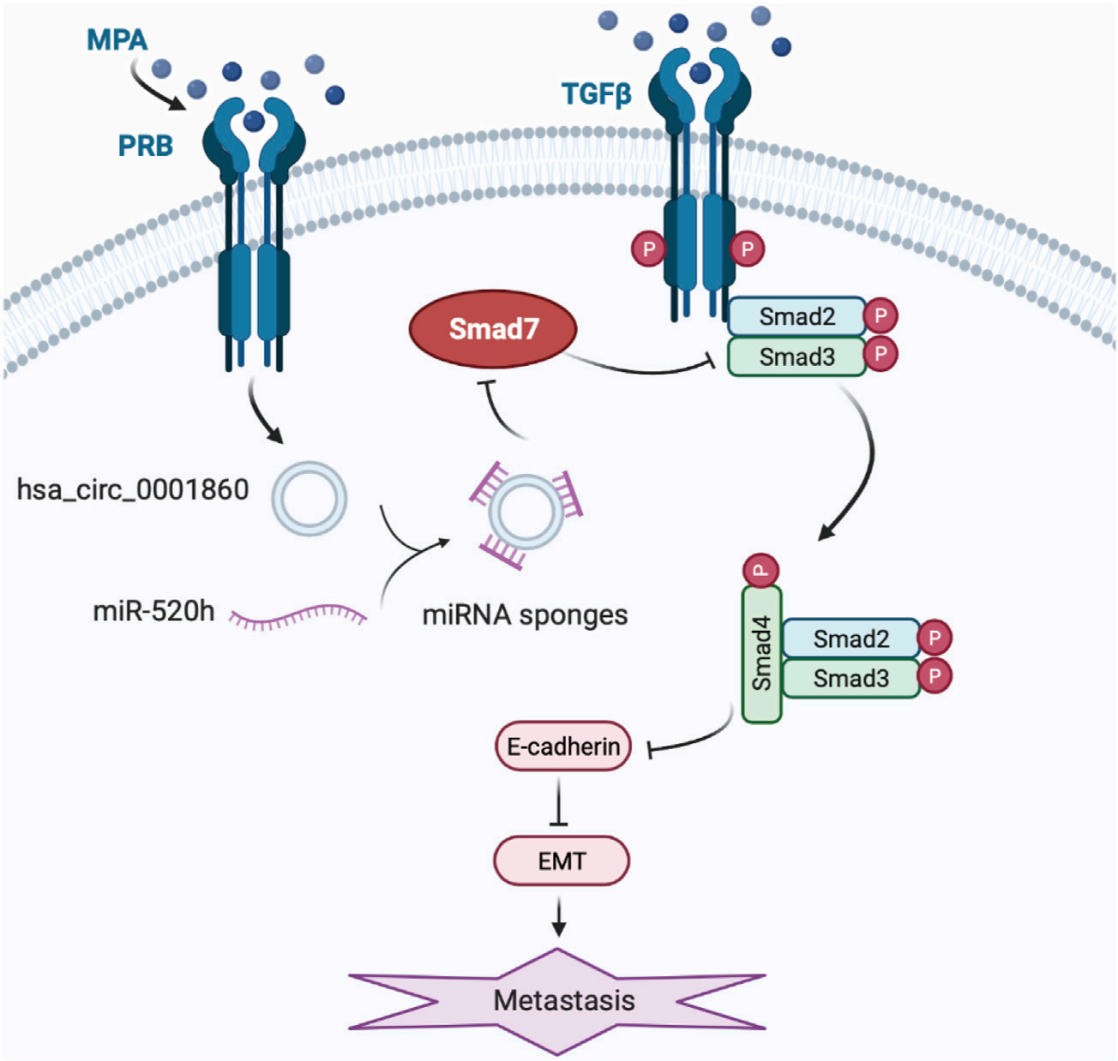
A working model for the role of hsa_circ_0001860 in EMT of EC cells treated with MPA. The expression upregulates in EC cells treated with MPA treatment induces increase of hsa_circ_0001860, which functions as a ceRNA for miR-520h, and regulates the expression and activity of Smad7, thus inhibits phosphorylation of Smad2/3, leading to the formation of Smad2/4 complex and nuclear translocation, and enhanced E-cadherin expression. Then, increased E-cadherin inhibits the migration and invasion and EC progression through EMT.

Based on the abovementioned studies, we demonstrated that downregulating miR-520 h reversed MPA resistance and inhibited MPA-induced migration and invasion in ISK-sh-circ_0001860 cells. Moreover, downregulation of hsa_circ_0001860 suppressed Smad7 protein expression, which could be reversed by the concurrent downregulation of miR-520 h. Therefore, our study presented a model of EMT process in EC cells treated with MPA, in which hsa_circ_0001860 may play a crucial role in EC metastasis and MPA resistance (Figure 7). However, there are some drawbacks in this study, and we have not yet discussed why the ISK cell treated with MPA leads to an increase in hsa_circ_0001860, which may be related to the expression of PRB. We speculated that PRB may regulate hsa_circ_0001860 expression in EC as a transcription factor. Moreover, we found that ISK^PRB−/−^ cells showed the same effect of MPA treatment when circRNA was overexpressed, which implied that hsa_circ_0001860 may affect the expression of PRB and form a feedback loop, but it needs further studies to explore and verify. The RNA pull-down and circRIP should also be done to confirm the interaction and the sponging effect between miR-520 h and hsa_circ_0001860. For the *in vivo* experiments, we did not use animal models of EC and measure the expression of hsa_circ_0001860 in blood plasma. We need also to measure more cancer tissues to validate the diagnostic performance of hsa_circ_0001860. We will address these issues in subsequent studies.

## Conclusion

In conclusion, we show that hsa_circ_0001860 plays an important role in the resistance of EC to MPA through miR-520h/Smad7 axis, and it could be developed into a novel marker and therapeutic target for MPA-resistant endometrial cancer.

## Data Availability

The datasets presented in this study can be found in online repositories. The names of the repository/repositories and accession number(s) can be found below: GSE 180424.

## Abbreviations

ATCC, American Type Culture Collection; BP, biological process; CC, cellular component; CCK8, cell counting kit-8; ceRNA, competing endogenous RNA; ciRNAs, circulation intronic RNA; ecircRNA, exotic circular RNA; circRNA, circular RNA; DMEM, Dulbecco’s modified Eagle medium; DMSO, dimethyl sulfoxide; EC, endometrial cancer; ECL, enhanced chemiluminescence; EiciRNA, exotic-intron circular RNA; EMT, epithelial-to-mesenchymal transition; FIGO, Federation International of Gynecology and Obstetrics; GO, Gene Ontology; lncRNA, long non-coding RNA; ISK, Ishikawa; KEGG, Kyoto Encyclopedia of Genes and Genomes; miRNA, microRNA; MPA, medroxyprogesterone acetate; MA, megestrol acetate; MF, molecular function; PR, progesterone receptor; PRB, progesterone receptor B; PVDF, polyvinylidene fluoride membrane; qRT-PCR, quantitative reverse transcription polymerase chain reaction; RNA-seq, high-throughput RNA sequencing; shRNA, short hairpin RNA; SD, standard deviation.
