# Supplementary material for: Hsa_Circ_0001860 Promotes Smad7 to Enhance MPA Resistance in Endometrial Cancer via miR-520h
Source: Front Cell Dev Biol. 2021 Nov 29;9:738189. doi: 10.3389/fcell.2021.738189 (PMC8666979; doi:10.3389/fcell.2021.738189)
Supplement: Supplementary file 1 [file DataSheet1.ZIP › Additional files/Additional file 11.docx]

**Additional file 12.** Sanger sequencing results of has_circ_0001860 were analyzed by Chromas.

1. **Gene: hsa_circ_0001860**
   1. **Splice site**：GAAGCTTC
   2. **Primers:**

F:GCTGGATGCTACTGGGATG

R:TGGGCATAATGAATTTGGCT

- 1. **PCR product sequence**

>hsa_circ_0001860

TTATAGAGGTCAGTTCAAGTGAAGAGGAAGAGAGCACCATTTCAGAAGGTGATAATGTGGAAAGCTGGATGCTACTGGGATGTGAAGTAGATGATAAAGATGATGATATCCTTCTCAACCTTGTGGGATGTGAAAACTCTGTTACTGAAGCTTCAAGGTTACTGACTTTTTATGATGTTTGGTGGCTATGAGACTATAGAAGCATACGAAGATGATCTTTATCGAGATGAGTCATCTAGTGAACTGAGTGTTGATAGTGAGGTGGAATTTCAACTCTATAGCCAAATTCATTATGCCCAA

- 1. **Result of sequencing：**

>F_2342.3F.ab1

ACGGGATAAGATGATGATATCCTTCTCACCTTGTGGGATGTGAAAACTCTGTTACTGAAGCTTCAAGGTTACTGACTTTTTATGATGTTTGGTGGCTATGAGACTATAGAAGCATACGAAGATGATCTTTATCGAGATGAGTCATCTAGTGAACTGAGTGTTGATAGTGAGGTGGAATTTCAACTCTATAGCCAAATTCATTATGCCCAA

- 1. **Sequencing Spectrum：**


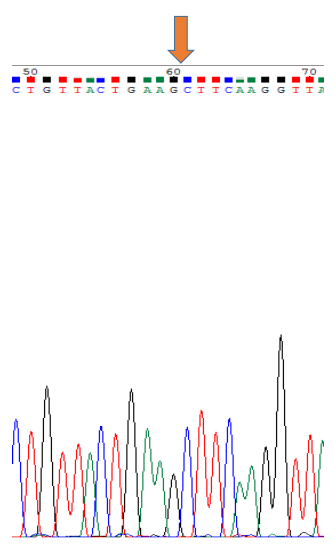


**1.6 Conclusion:** The splice site of gene hsa_circ_0001860 can be found in PCR product sequence indicating the clone sequence is the target sequence.
