# Supplementary material for: Hsa_Circ_0001860 Promotes Smad7 to Enhance MPA Resistance in Endometrial Cancer via miR-520h
Source: Front Cell Dev Biol. 2021 Nov 29;9:738189. doi: 10.3389/fcell.2021.738189 (PMC8666979; doi:10.3389/fcell.2021.738189)
Supplement: Supplementary file 1 [file DataSheet1.ZIP › Additional files/Additional file 2-Table S2.docx]

**Additional file 2: Table S2.** Sequence of qPCR primers for the detection of circRNAs, miRNAs and mRNAs.

| **number** | **CircRNA-ID** | **circBase-ID** | **Primers** (5’-3’) |
| --- | --- | --- | --- |
| circRNA-1 | chr10:112723883-112745523+ | hsa_circ_0020028 | Forward: CCAACCTTGACTTGCAGC  Reverse: TGCCCATTTCTGATCATTCA |
| circRNA-2 | chr14:62187100-62188541+ | hsa_circ_0006393 | Forward: TCCATGTGACCATGAGGAAA  Reverse: GAGATCTGGCTGCATCTCG |
| circRNA-3 | chr13:20534098-20568059+ | / | Forward: TGAATGTGGCAGGAGACG  Reverse: AGGGCTGAAGGCGATTCT |
| circRNA-4 | chr20:39721112-39729993+ | hsa_circ_0115215 | Forward: GTGGAAAGAAGTCCGGCA  Reverse: TGGTGGGGCAAATACTGG |
| circRNA-5 | chr10:70196768-70229920- | hsa_circ_0018524 | Forward: CATGGTGCCATACCTGTCA  Reverse: CCAGGCGCTTTTCACAGT |
| circRNA-6 | chr3:56626998-56628056+ | hsa_circ_0001313 | Forward: TGCTCTCTTGGACCCAGC  Reverse: GAAAGGGTGCTCCAGCAGT |
| circRNA-7 | chr9:37126309-37126939+ | hsa_circ_0001860 | Forward: GCTGGATGCTACTGGGATG  Reverse: TGGGCATAATGAATTTGGCT |
| circRNA-8 | chr18:74561482-74583781+ | hsa_circ_0001993 | Forward: CACGTATTCGTGTCCGCA  Reverse: TGGCGTTGAAACTGGGAT |
| circRNA-9 | chr2:74834182-74867454- | / | Forward: CGTGGACTCAGCGTCTCC  Reverse: CCAGTAGTTCGCCCAGGA |
| circRNA-10 | chr7:157009561-157024021+ | hsa_circ_0133805 | Forward: CCCAACCAGGGGTTCTTT  Reverse: CAGCCAGTGGTTTGGAGG |
| circRNA-11 | chr4:88104355-88116842- | hsa_circ_0006866 | Forward: TCCATTCGGACTACCCCA  Reverse: CCCACTCAACTGCCATTGT |
| circRNA-12 | chr16:14674731-14721193- | hsa_circ_0105045 | Forward: ACGAGGCAGGCTACGATG  Reverse: TGCAAAGGCCAAACTGAA |
| circRNA-13 | chr18:9195549-9221997+ | hsa_circ_0046843 | Forward: GGAGCGTCCAGTGGATGTA  Reverse: TCCTTGCTCACATCACTTCG |
| circRNA-14 | chr2:239090706-239093928- | hsa_circ_0001116 | Forward: CTCGGAGATAGTCGGGCA  Reverse: TCGAATTCCTCCATGTCCA |
| circRNA-15 | chr10:126097111-126100769- | hsa_circ_0008898 | Forward: GGCTGGAGAGACTGCCTG  Reverse: GGAGGGCCTTGGACTGTT |
| circRNA-16 | chr6:108225833-108246136- | hsa_circ_0009144 | Forward: AATAGTAATGGCCCGGAACC  Reverse: GAACAATGCCCATCCTGC |
| circRNA-17 | chr4:178274462-178274882+ | hsa_circ_0001459 | Forward: AAAAGCTGCAACCCTGGA  Reverse: CCAAATGCAGTTTTTCTGTTG |
| circRNA-18 | chr15:55835782-55837423- | / | Forward: TTAAGTTTCAGTAGTTAGCTGT  Reverse: TTCCTCATCTGCAATATGTCC |
| circRNA-19 | chr4:151719233-151738409- | hsa_circ_0008618 | Forward: AGCCATGGACAAGAACTGCT  Reverse: AAGGTGGAAGGTGAGACTGC |
| circRNA-20 | chr18:45391430-45423180- | hsa_circ_0000847 | Forward: TATTCCAGAAACGCCACCTC  Reverse: TTCCATCCCAGCAGTCTCTT |

Note: / means a novel circRNA which has not been in circBase.

| **Primer sequence** |  |  |
| --- | --- | --- |
| actin | Forward | CATGTACGTTGCTATCCAGGC |
| actin | Reverse | CTCCTTAATGTCACGCACGAT |
| U6 | Forward | CTCGCTTCGGCAGCACA |
| U6 | Reverse | AACGCTTCACGAATTTGCGT |
| E-cadherin | Forward | ATTCTGATTCTGCTGCTCTTG |
| E-cadherin | Reverse | AGTAGTCATAGTCCTGGTCTT |
| N-cadherin | Forward | CTCCTATGAGTGGAACAGGAACG |
| N-cadherin | Reverse | TTGGATCAATGTCATAATCAAGTGCTGTA |
| Smad7 | Forward | TTCCTCCGCTGAAACAGGG |
| Smad7 | Reverse | CCTCCCAGTATGCCACCAC |
| GAPDH | Forward | GGAGCGAGATCCCTCCAAAAT |
| GAPDH | Reverse | GGCTGTTGTCATACTTCTCATGG |
| hsa-miR-520h | Forward | TGCGCACAAAGTGCTTCCCTTT |
| Universal 5’ primer |  | GCGAGCACAGAATTAATACGAC |
