# Supplementary material for: Hsa_Circ_0001860 Promotes Smad7 to Enhance MPA Resistance in Endometrial Cancer via miR-520h
Source: Front Cell Dev Biol. 2021 Nov 29;9:738189. doi: 10.3389/fcell.2021.738189 (PMC8666979; doi:10.3389/fcell.2021.738189)
Supplement: Supplementary file 1 [file DataSheet1.ZIP › Additional files/Additional file 10-Table S7.docx]

**Additional file 11: Table S7.** qPCR analysis of gene expression level changes before and after treated with R Nase R.

| Gene | Rnase R | Mock | Ct(RnaseR)-Ct(Mock) | Fold Enrichment | **Relative Ratio** | |
| --- | --- | --- | --- | --- | --- | --- |
|  |  |  |  |  | Mock | Rnase R |
| GAPDH | 30.777 | 17.474 | 13.303 | 10107.434 | 1.000 | 0.000 |
| chr9:37126309-37126939+  (hsa_circ_0001860) | 24.553 | 23.682 | 0.871 | 1.829 | 1.000 | 0.547 |
