# Supplementary material for: Hsa_Circ_0001860 Promotes Smad7 to Enhance MPA Resistance in Endometrial Cancer via miR-520h
Source: Front Cell Dev Biol. 2021 Nov 29;9:738189. doi: 10.3389/fcell.2021.738189 (PMC8666979; doi:10.3389/fcell.2021.738189)
Supplement: Supplementary file 1 [file DataSheet1.ZIP › Additional files/Additional file 13-Table S8.docx]

**Additional file 14: Table S8.** The minimum free energy hybridization scores for CircRNA-miRNA pairs were calculated by the RNAhybrid.

| position | circRNA-miRNA pair (has_circ_0001860-miR-520h) | MFE* (kcal/mol) | P-value |
| --- | --- | --- | --- |
| 285 | **Target: 5' gUC-AGAGGUCAUCACUUUGUc 3'** **\|\| \|:\|\|\|  \| \|\|\|\|\|\|\|\|** **miRNA : 3'ugAGAUUUCCCUUCGUGAAACA 5'** | -19.7 | <0.001 |
| 500 | **Target: 5' gUGAAGAGGAAGAGAGCACc 3'** **\|:\|\|\| \|\|\|    \|\|\|\|\|** **miRNA : 3'ugagAUUUC-CCU----UCGUGaaaca 5'** | -18.7 | <0.001 |
| 163 | **Target: 5' gUCAUCAGGGAGGAAGAGCAUg 3'** **\|\| \|\|\|\|\|     \|\|\|\|:** **miRNA : 3'ugAGAUUUCCCU-----UCGUGaaaca 5'** | -17.8 | <0.001 |
| 598 | **Target: 5'aAC-CUUGUGGGAUGUGAAAACUCUGUu 3'** **\|\| \|\| \|\|\|\| \|:    \|\|\| \|\|\|** **miRNA : 3' UGAGAUUUCCCUUCG----UGAAACA 5'** | -16.7 | <0.001 |
| 343 | **Target: 5'. gUUAGAGUUCAAGCACa 3'** **:\|\|:\|\| \|\|\|\|\|\|**  **miRNA : 3' ugaGAUUUCCC-UUCGUGaaaca 5'** | -16.7 | <0.001 |
