# Supplementary material for: Hsa_Circ_0001860 Promotes Smad7 to Enhance MPA Resistance in Endometrial Cancer via miR-520h
Source: Front Cell Dev Biol. 2021 Nov 29;9:738189. doi: 10.3389/fcell.2021.738189 (PMC8666979; doi:10.3389/fcell.2021.738189)
Supplement: Supplementary file 1 [file DataSheet1.ZIP › Additional files/Additional file 4-Table S4.docx]

**Additional file 4: Table S4.** Detailed information of antibody used in this study.

| Antibody | Supplier | Catalogue | Primary/secondary | Host | Dilution |
| --- | --- | --- | --- | --- | --- |
| GAPDH | Abcam | ab181602 | P | Rabbit | 1:10000 |
| ACTIN | CST | 4970 | P | Rabbit | 1:1000 |
| E-cadherin | CST | 3195 | P | Rabbit | 1:1000 |
| N-cadherin | Abcam | ab76011 | P | Rabbit | 1:1000 |
| Smad7 | Abcam | ab90086 | P | Rabbit | 1:1000 |
| Smad2/3 | CST | 8685 | P | Rabbit | 1:1000 |
| P-smad2/3 | CST | 8828 | P | Rabbit | 1:1000 |
| Goat anti-rabbit IgG | Abcam | ab6721 | S | Goat | 1:10000 |
