# Supplementary material for: Hsa_Circ_0001860 Promotes Smad7 to Enhance MPA Resistance in Endometrial Cancer via miR-520h
Source: Front Cell Dev Biol. 2021 Nov 29;9:738189. doi: 10.3389/fcell.2021.738189 (PMC8666979; doi:10.3389/fcell.2021.738189)
Supplement: Supplementary file 1 [file DataSheet1.ZIP › Additional files/Additional file 7-Table S5.docx]

**Additional file 7: Table S5.** List of host genes clustered.

| **circRNAs ID** | **hosting genes** | **Regulation** |
| --- | --- | --- |
| chr2:106774514-106782539- | UXS1 | up |
| chr4:151719233-151738409- | LRBA | up |
| chr6:108225833-108246136- | SEC63 | up |
| chr10:96201653-96234540+ | TBC1D12 | up |
| chr14:31346778-31349940+ | COCH | up |
| chr7:129813472-129818367- | TMEM209 | up |
| chr3:47663697-47719801- | SMARCC1 | up |
| chr10:126727566-126799662- | CTBP2 | up |
| chr7:157009561-157024021+ | UBE3C | up |
| chr10:70218861-70229920- | DNA2 | up |
| chr1:11133991-11137005- | EXOSC10 | up |
| chrX:14861782-14877456- | FANCB | up |
| chr4:154315414-154318485+ | MND1 | up |
| chr15:85657104-85664245+ | PDE8A | up |
| chr2:10928823-10930959- | PDIA6 | up |
| chrX:24828015-24861794+ | POLA1 | up |
| chr3:56626998-56628056+ | CCDC66 | up |
| chr13:20534098-20568059+ | ZMYM2 | up |
| chr15:51855578-51868378- | DMXL2 | up |
| chr17:57647879-57650596+ | DHX40 | up |
| chr3:180665653-180667131+ | FXR1 | up |
| chr19:9763629-9764557- | ZNF562 | up |
| chr8:131370263-131374017- | ASAP1 | up |
| chr15:81271107-81274523- | G028499 | up |
| chr1:32495899-32498935+ | KHDRBS1 | up |
| chr10:128859932-128908618+ | DOCK1 | up |
| chr15:55835782-55837423- | PYGO1 | up |
| chr9:33941647-33953472- | UBAP2 | up |
| chr18:76856476-76914555+ | ATP9B | up |
| chr15:51827860-51839605- | DMXL2 | up |
| chr8:42259306-42260979+ | VDAC3 | up |
| chr5:112889464-112899189+ | YTHDC2 | up |
| chr3:169854207-169896726- | PHC3 | up |
| chr6:116966877-116982009- | ZUFSP | up |
| chr4:178274462-178274882+ | NEIL3 | up |
| chr2:239090706-239093928- | ILKAP | up |
| chr2:99786013-99787892- | MITD1 | up |
| chr7:100410369-100410830- | EPHB4 | up |
| chr10:112723883-112745523+ | SHOC2 | up |
| chr2:128922304-128928858+ | UGGT1 | up |
| chr9:37126309-37126939+ | ZCCHC7 | up |
| chr19:47421745-47440665+ | ARHGAP35 | up |
| chr3:168830575-168861620- | MECOM | up |
| chr13:42439872-42442613- | VWA8 | up |
| chr16:68155890-68160513+ | NFATC3 | up |
| chr12:70193989-70195501+ | RAB3IP | up |
| chr14:39648295-39648666+ | PNN | down |
| chr22:25771780-25771975- | LRP5L | down |
| chr4:88104355-88116842- | KLHL8 | down |
| chr10:70196768-70229920- | DNA2 | down |
| chr3:122928046-122978436+ | SEC22A | down |
| chr6:155095123-155116273+ | SCAF8 | down |
| chr11:117023157-117034608+ | PAFAH1B2 | down |
| chr5:619105-620376+ | CEP72 | down |
| chr2:159992705-160007067+ | TANC1 | down |
| chr19:45766371-45766625+ | MARK4 | down |
| chr11:93523740-93535138+ | MED17 | down |
| chr5:43122141-43162033+ | ZNF131 | down |
| chr11:22242643-22261230+ | ANO5 | down |
| chrX:3735586-3747433- | LOC389906 | down |
| chr10:126097111-126100769- | OAT | down |
| chrX:102081897-102160665+ | LINC00630 | down |
| chr18:9195549-9221997+ | ANKRD12 | down |
| chr14:62187100-62188541+ | HIF1A | down |
| chr13:111274563-111276626+ | CARKD | down |
| chr19:34922766-34955036+ | UBA2 | down |
| chr16:14674731-14721193- | PARN | down |
| chr1:156303338-156304709- | CCT3 | down |
| chr12:12300300-12303972- | LRP6 | down |
| chr17:74283273-74301022- | QRICH2 | down |
| chr11:85718585-85742653- | PICALM | down |
| chr2:74834182-74867454- | M1AP | down |
| chr13:21955573-21965993- | ZDHHC20 | down |
| chr4:151656410-151729550- | LRBA | down |
| chr10:70719562-70720005+ | DDX21 | down |
| chr18:74561482-74583781+ | ZNF236 | down |
| chr20:39721112-39729993+ | TOP1 | down |
| chr9:88211277-88248289- | AGTPBP1 | down |
| chr14:56114743-56115588+ | KTN1 | down |
| chr10:120488807-120489922- | CACUL1 | down |
| chr6:146209156-146216113- | SHPRH | down |
| chr18:45391430-45423180- | SMAD2 | down |
| chr2:203620261-203624076+ | FAM117B | down |
| chr3:168830575-168849319- | MECOM | down |
| chr3:196533450-196534785+ | PAK2 | down |
| chr7:22306583-22357656- | RAPGEF5 | down |
| chr3:113207792-113225452- | SPICE1 | down |
