# Supplementary material for: Hsa_Circ_0001860 Promotes Smad7 to Enhance MPA Resistance in Endometrial Cancer via miR-520h
Source: Front Cell Dev Biol. 2021 Nov 29;9:738189. doi: 10.3389/fcell.2021.738189 (PMC8666979; doi:10.3389/fcell.2021.738189)
Supplement: Supplementary file 1 [file DataSheet1.ZIP › Additional files/Additional file 8-Table S6.docx]

**Additional file 9: Table S6.** Twenty differentially expressed circRNAs were validated by qRT-PCR.

| number | CircRNA-ID | logFC | PValue | circBase-ID | GeneName |
| --- | --- | --- | --- | --- | --- |
| circRNA-1 | chr10:112723883-112745523+ | 2.871 | 0.029 | hsa_circ_0020028 | SHOC2 |
| circRNA-2 | chr14:62187100-62188541+ | -3.883 | 0.035 | hsa_circ_0006393 | HIF1A |
| circRNA-3 | chr13:20534098-20568059+ | 3.920 | 0.040 | / | ZMYM2 |
| circRNA-4 | chr20:39721112-39729993+ | -3.038 | 0.028 | hsa_circ_0115215 | TOP1 |
| circRNA-5 | chr10:70196768-70229920- | -4.157 | 0.020 | hsa_circ_0018524 | DNA2 |
| circRNA-6 | chr3:56626998-56628056+ | 3.957 | 0.031 | hsa_circ_0001313 | CCDC66 |
| circRNA-7 | chr9:37126309-37126939+ | 2.767 | 0.044 | hsa_circ_0001860 | ZCCHC7 |
| circRNA-8 | chr18:74561482-74583781+ | -3.260 | 0.008 | hsa_circ_0001993 | ZNF236 |
| circRNA-9 | chr2:74834182-74867454- | -3.803 | 0.046 | / | M1AP |
| circRNA-10 | chr7:157009561-157024021+ | 4.104 | 0.030 | hsa_circ_0133805 | UBE3C |
| circRNA-11 | chr4:88104355-88116842- | -4.279 | 0.015 | hsa_circ_0006866 | KLHL8 |
| circRNA-12 | chr16:14674731-14721193- | -3.851 | 0.038 | hsa_circ_0105045 | PARN |
| circRNA-13 | chr18:9195549-9221997+ | -3.917 | 0.034 | hsa_circ_0046843 | ANKRD12 |
| circRNA-14 | chr2:239090706-239093928- | 3.071 | 0.017 | hsa_circ_0001116 | ILKAP |
| circRNA-15 | chr10:126097111-126100769- | -3.955 | 0.033 | hsa_circ_0008898 | OAT |
| circRNA-16 | chr6:108225833-108246136- | 4.390 | 0.012 | hsa_circ_0009144 | SEC63 |
| circRNA-17 | chr4:178274462-178274882+ | 3.170 | 0.010 | hsa_circ_0001459 | NEIL3 |
| circRNA-18 | chr15:55835782-55837423- | 3.824 | 0.049 | / | PYGO1 |
| circRNA-19 | chr4:151719233-151738409- | 4.472 | 0.009 | hsa_circ_0008618 | LRBA |
| circRNA-20 | chr18:45391430-45423180- | -2.020 | 0.025 | hsa_circ_0000847 | SMAD2 |

Note: / means a novel circRNA which has not been in circBase.
