# Supplementary material for: Hsa_Circ_0001860 Promotes Smad7 to Enhance MPA Resistance in Endometrial Cancer via miR-520h
Source: Front Cell Dev Biol. 2021 Nov 29;9:738189. doi: 10.3389/fcell.2021.738189 (PMC8666979; doi:10.3389/fcell.2021.738189)
Supplement: Supplementary file 1 [file DataSheet1.ZIP › Additional files/Additional file 1-Table S1.docx]

**Additional file 1: Table S1.** ShRNA sequences used in this study.

| **shRNAs** |  |
| --- | --- |
| shPRB | GCTGTAAGGTCTTCTTTAA |
| circ_0001860 sh circ-1 | ACTCTGTTACTGAAGCTTCAA |
| circ_0001860 sh circ-2 | TTACTGAAGCTTCAAGGTTAC |
| sh-Smad7 | AGGACGCTGTTGGTACACA |
| sh-NC | TTCTCCGAACGTGTCACGT |
