# Supplementary material for: Hsa_Circ_0001860 Promotes Smad7 to Enhance MPA Resistance in Endometrial Cancer via miR-520h
Source: Front Cell Dev Biol. 2021 Nov 29;9:738189. doi: 10.3389/fcell.2021.738189 (PMC8666979; doi:10.3389/fcell.2021.738189)
Supplement: Supplementary file 1 [file DataSheet1.ZIP › Additional files/Additional file 3-Table S3.docx]

**Additional file 3: Table S3.** MiRNA mimics, and inhibitors sequences used in this study.

| **mimics and inhibitors** |  |
| --- | --- |
| mimics miR-520h-sense | ACAAAGUGCUUCCCUUUAGAGU |
| mimics miR-520h-antisense | UCUAAAGGGAAGCACUUUGUUU |
| mimics nc-sense | UUCUCCGAACGUGUCACGUTT |
| mimics nc-antisense | ACGUGACACGUUCGGAGAATT |
| miR-520h inhibitor | ACUCUAAAGGGAAGCACUUUGU |
| Inhibitor nc | CAGUACUUUUGUGUAGUACAA |
