# Supplementary figures and images for: Hsa_Circ_0001860 Promotes Smad7 to Enhance MPA Resistance in Endometrial Cancer via miR-520h
Source: Front Cell Dev Biol. 2021 Nov 29;9:738189. doi: 10.3389/fcell.2021.738189 (PMC8666979; doi:10.3389/fcell.2021.738189)

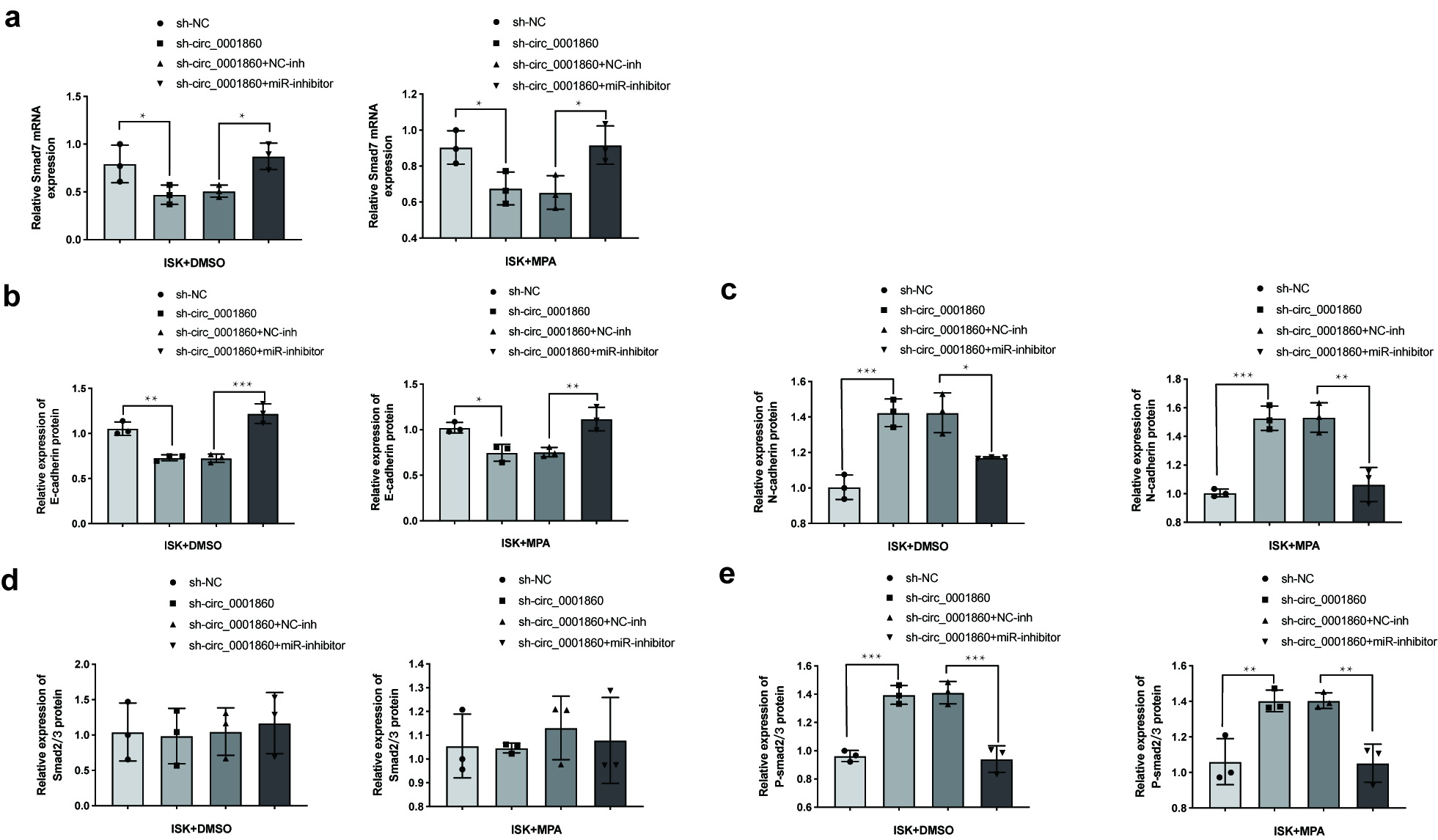

Supplement: Supplementary file 1 [file DataSheet1.ZIP › Additional files/Additional file 14-Figure S5.tif]

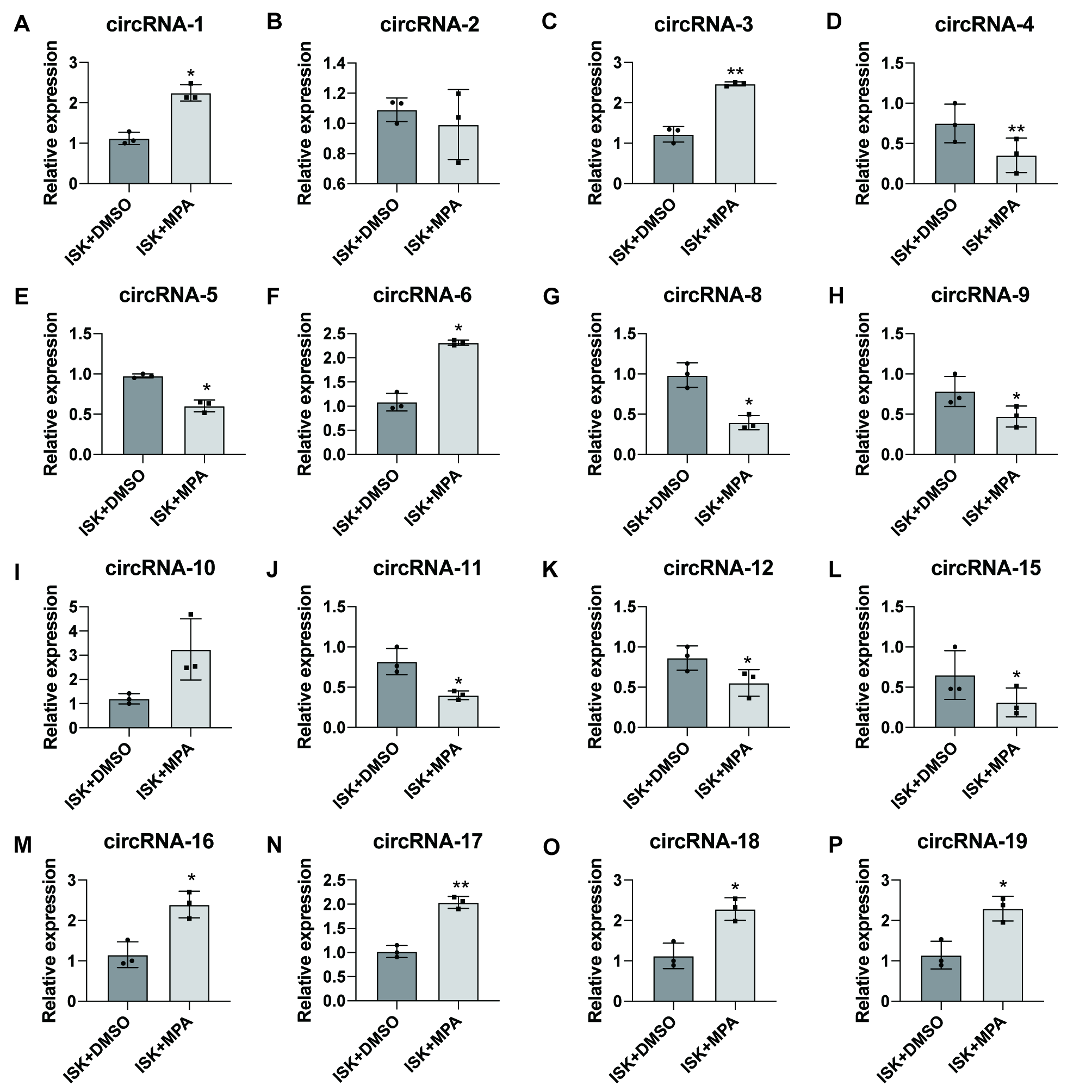

Supplement: Supplementary file 1 [file DataSheet1.ZIP › Additional files/Additional file 9-Figure S3.tif]

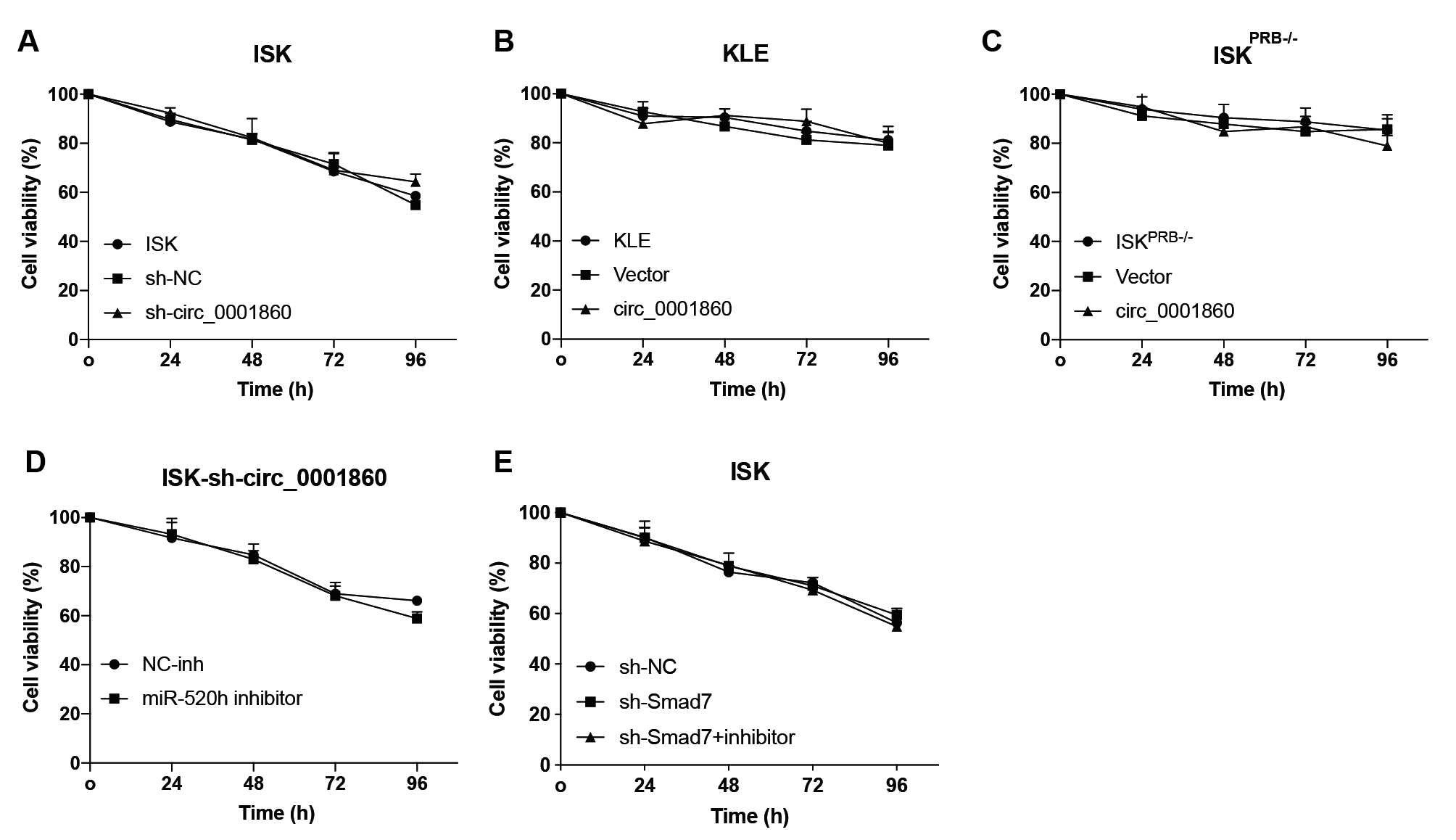

Supplement: Supplementary file 1 [file DataSheet1.ZIP › Additional files/Additional file 12-Figure S4.tif]

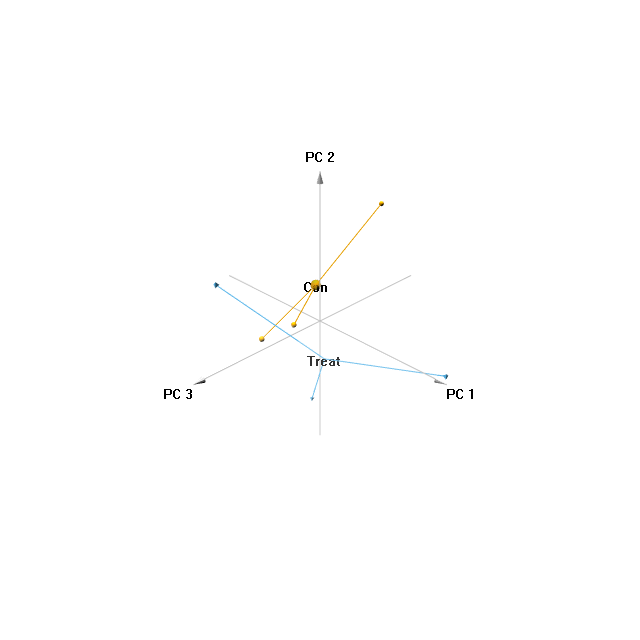

Supplement: Supplementary file 1 [file DataSheet1.ZIP › Additional files/Additional file 5-Figure S1.tiff]

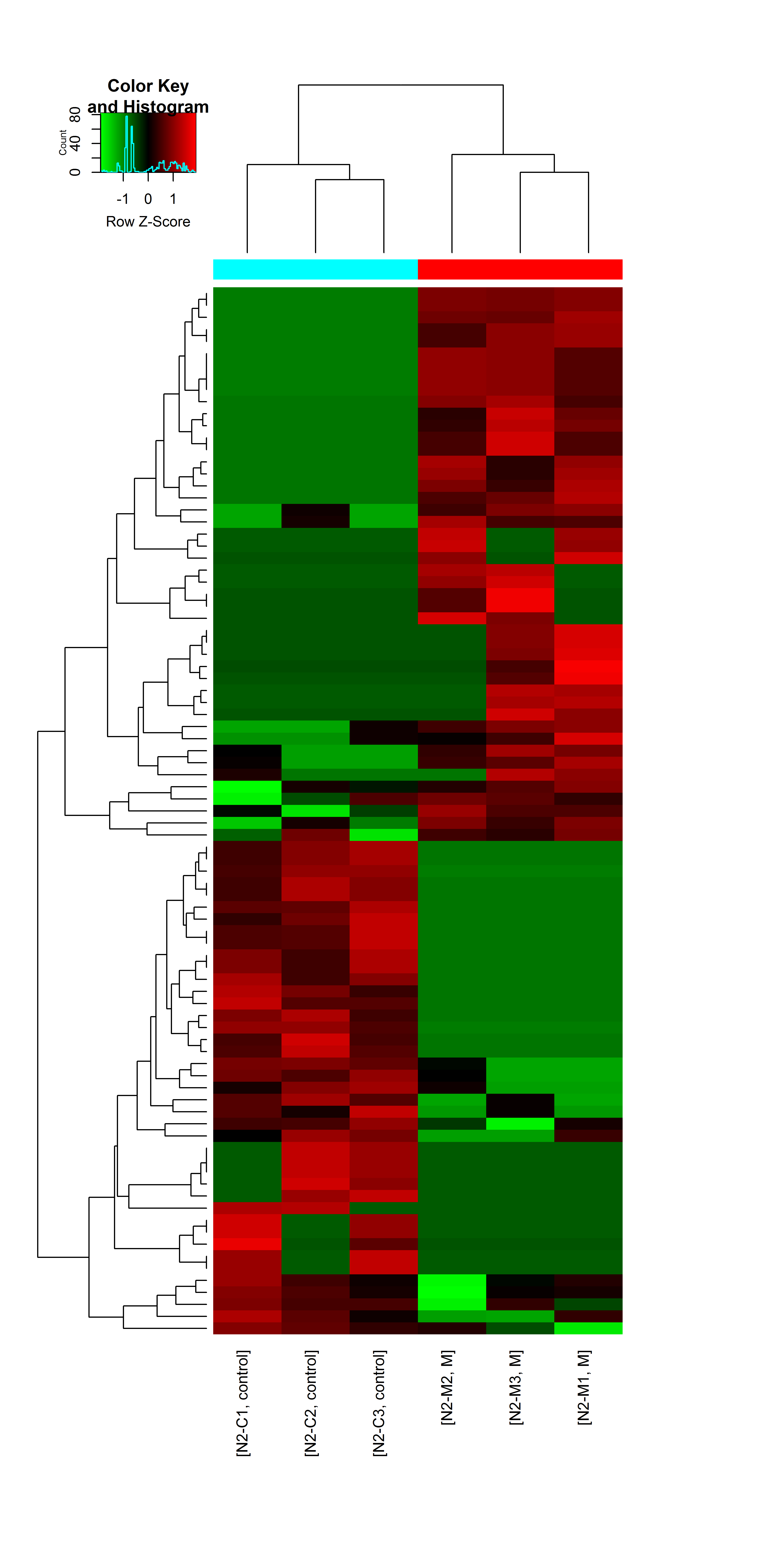

Supplement: Supplementary file 1 [file DataSheet1.ZIP › Additional files/Additional file 6-Figure S2.tif]

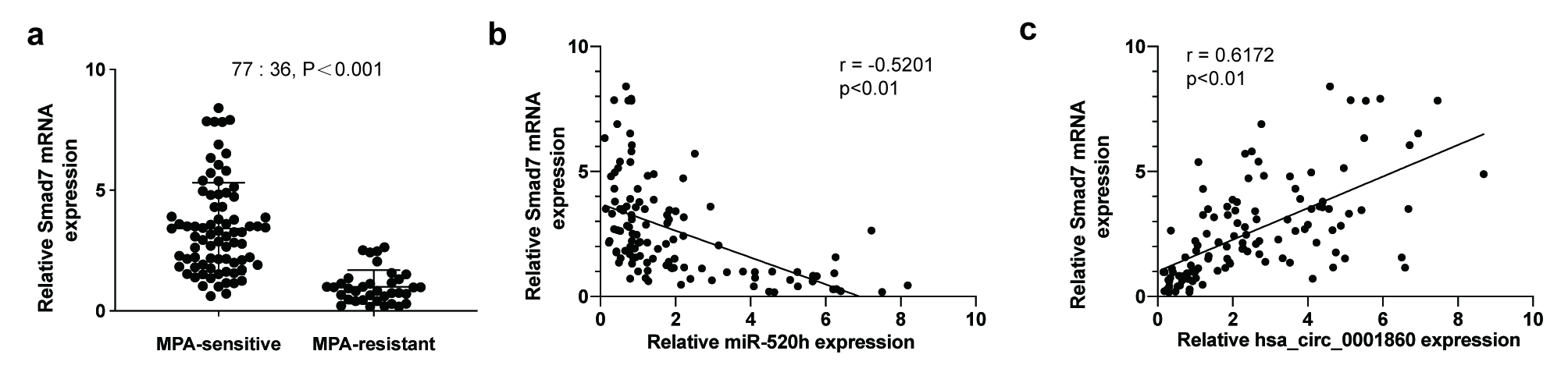

Supplement: Supplementary file 1 [file DataSheet1.ZIP › Additional files/Additional file 15-Figure S6.tif]
